# Predictors of self-care in patients with cancer treated with oral anticancer agents: A systematic review

**DOI:** 10.1371/journal.pone.0307838

**Published:** 2024-09-24

**Authors:** Silvia Ucciero, Federica Lacarbonara, Angela Durante, Francesco Torino, Izabella Uchmanowicz, Ercole Vellone, Marco Di Nitto

**Affiliations:** 1 Department of Biomedicine and Prevention, Tor Vergata University of Rome, Rome, Italy; 2 School of Advanced Studies Sant’Anna, Health Science Center, Pisa, Italy; 3 Department of Systems Medicine, Medical Oncology, Tor Vergata University of Rome, Rome, Italy; 4 Department of Nursing and Obstetrics, Wroclaw Medical University, Wroclaw, Poland; 5 Department of Health Sciences, University of Genoa, Genoa, Italy; Federal University Dutse, NIGERIA

## Abstract

**Background:**

In the last two decades, the use of oral anticancer agents (OAAs) has increased in cancer patients. Despite this, patients and their caregivers face some challenging issues (side effects, drug-to-drug interactions, etc.) related to OAA administration. The three dimensions of self-care by Riegel et al., self-care maintenance (i.e., stability of patient condition), self-care monitoring (i.e., detection of side effects), and self-care management (i.e., management of side effects), may be implemented to avoid negative outcomes. However, knowledge of self-care determinants is necessary to recognise people at risk of poor self-care behaviours.

**Aims:**

Determine which are the predictors of self-care maintenance, self-care monitoring and self-care management in patients with cancer taking OAA.

**Methods:**

A systematic review with narrative synthesis was conducted. We included studies on adult patients with cancer using any kind of oral anticancer agent and describing a predictor of self-care. The search was performed on PubMed, CINAHL/PsycINFO, and Web of Science.

**Results:**

Of 3,061 records, 45 studies were included in this review. Forty-six predictors organised into 14 categories were identified. In general, all studies focused only on adherence, considered as a self-care maintenance component, and none of them focused on other dimensions of self-care. The predictors of OAA adherence most reported were age, side effects, and socioeconomic factors (e.g., insurance status, and annual income).

**Conclusions:**

This systematic review highlighted the literature gap on the analysis of determinants of self-care behaviours in patients taking OAAs. This element could be a starting point for future research that can provide elements to support the oncology nursing research agenda, aimed at recognising patients at risk of poor self-care.

## 1. Introduction

Oral anticancer agents (OAA) are antineoplastic medications administered orally that can be classified according to their chemical composition and mechanism of action. The OAAs are cytotoxic agents, targeted therapies, or hormone therapies. In the last two decades, the use of OAAs has increased due to the considerable advantages of OAAs [1, 2], including the possibility that patients continue to live their everyday lives, take their treatment at home, avoid problems related to intravenous administrations of chemotherapy, hospitalizations, and the related infection risk [3], with reduced physical and psychological impact of cancer treatment, and have greater feeling of control over the treatment [4]. However, despite the ease of OAA administration, some issues can negatively impact patient’s quality of life and disease course. Previous studies reported that patients sometimes do not respect OAA regimens either in terms of poor adherence (e.g., due to comorbidities, mental disorders, adverse events or medicines costs), leading to possible reductions in therapeutic effects of treatment [5], or in terms of over adherence, sometimes due to the forgetfulness of having already taken a dose, with the risk of adverse events that must be detected and managed at home [6, 7]. These risks can be prevented if patients taking OAAs receive education on self-care, which does not include only pharmacological adherence but also behaviour change [8, 9].

According to the theory of self-care in chronic illness by Riegel et al [10], self-care is a pull of behaviours aimed at maintaining and managing chronic condition. Self-care behaviours are grouped into three dimensions: self-care maintenance (i.e., behaviours performed to keep psychophysical state stable), self-care monitoring (i.e., behaviours performed for monitoring signs and symptoms), and self-care management (i.e., behaviours put in place in case of signs and symptoms of illness exacerbation). These three dimensions are considered fundamental also for cancer patients treated with OAA who need to implement self-care 1) to maintain stable disease condition and ensure proper adherence to OAA (self-care maintenance); 2) to be able to recognise any sign/symptom of decompensation or medication side effects (self-care monitoring), and 3) to manage promptly any sign/symptom that could occur (self-care management) [11].

Many studies have identified predictors and outcomes of self-care in chronic diseases, particularly in patients with heart failure [12] and type 2 diabetes mellitus [13]. From the study by Jaarsma et al., it was highlighted that knowledge, skills and experience of the disease, or even motivation, personal beliefs, habits or lifestyles, cognitive functions, and functional status can affect the patient’s level of self-care with heart failure [14]. The role of cognitive functions and functional status is also underlined in previous studies, according to which mental and cognitive functions are closely related to patient participation in self-care activities such as, for example, buying and preparing healthy food, being able to understand simple instructions on how to maintain a healthy lifestyle, or even being able to climb or descend stairs [10, 15]. Another study [13] reported that cognitive functions and sociodemographic factors (e.g., sex, age, marital status, and social support) were determinants of self-care in patients with type 2 diabetes mellitus.

For patients on OAAs, the predictors of self-care have not yet been explored. There is only sparse evidence regarding this topic and most of the literature focused on medication adherence [16, 17]. Medication adherence is an essential dimension in cancer care, but it can be considered only one part of the self-care behaviours the patient should put in place to manage adequately their disease. Moreover, a recent study [18], “the Situation-Specific Theory of Heart Failure Self-Care”, that is an update of the more general middle range Theory of Self-care in Chronic Illness by Riegel et al. [10], highlighted that self-care behaviours can be related to factors that can be grouped in three themes: person (e.g., age, health literacy, etc.), problem (e.g., sleep disorders, symptoms, etc.), and environment (e.g., the built environment, social support, etc.) factors.

The “Person” theme refers to the adult with Heart Failure; “Problem” theme refers to the physical and emotional consequences of the diagnosis; while the “Environment” theme refers to the setting in which the person is dealing with the Heart Failure diagnosis [18].

Moreover, in the same study it was reported that for heart failure patients only person-related factors were adequately explored, with sparse knowledge on problem- and environment-related factors, guiding future research on literature gaps.

It is very important for healthcare providers and, above all, for nurses to know predictors of self-care for patients with OAA to highlight obstacles or facilitators to the patient’s participation and their caregivers in the development of self-care [14].

Therefore, the objective of this review was to identify predictors of self-care maintenance, monitoring and management [10] in patients with cancer on OAAs.

## 2. Materials and methods

### 2.1 Study design

A systematic review with narrative synthesis was conducted [19]. Due to the heterogeneity of the selected studies, a meta-analysis could not be performed. The protocol was registered in the PROSPERO database on 02/13/2022 (CRD42022299684, available from https://www.crd.york.ac.uk/prospero/). The Statement of Preferred Reporting Items for Systematic Reviews and Meta-Analysis (PRISMA) of the systematic review was used to report results [20].

### 2.2 Search strategy

A systematic search strategy was used to search the following databases: PubMed, Cumulative Index of Nursing and Allied Health (CINAHL), Web of Science, and PsycINFO. In all databases, the search strategy was used using MeSH terms (Medical Subject Headings) and free text search terms. Due to the specific search characteristics of each database, the search strategy was first used in the PubMed database and then adapted in each database according to the purpose of the study and the inclusion criteria. In addition, a hand search was conducted to find other relevant articles.

Keywords selected were related to self-care (eg, ‘self-care’, ‘self-management’ and ‘adherence’, ‘self-monitoring’), anticancer therapy (eg, ‘antineoplastic agent’, ‘oncolytic agent’ and ‘targeted drugs’), predictors and outcomes (‘predictor’, ‘self-care determinants’, ‘outcomes’) and cancer (e.g., ‘neoplasm’, ‘tumour’ and ‘cancer’). The databases were searched from 2014 until the end of July 2023. The publication date limit was set to catch the most updated literature on this topic. Moreover, about what stated in the protocol, only studies that considered predictors of self-care were considered in this article, whereas outcomes will be reported in a subsequent publication.

The complete search strategy is available as supplementary material (Table S1A in S1 Appendix).

### 2.3 Eligibility criteria

The following eligibility criteria were adopted in this review: a) studies conducted in adult patients (≥ 18 years) with solid cancer at any anatomical site, excluding melanoma, receiving any kind of OAA (that is, systemic anticancer therapy, targeted therapy, hormonal therapy, etc.); b) studies reporting self-care predictors according to our adopted framework [10, 21, 22]; c) articles in English, Italian and Spanish; and d) articles reporting primary quantitative studies. All observational studies, such as cohort, case-control, cross-sectional and longitudinal studies, non-randomised controlled trials (quasi-experimental studies), and randomised controlled trials (RCT) were included. The following exclusion criteria were used: a) articles reporting studies in patients treated with oral *and* intravenous anticancer drugs; b) articles addressing the point of view of healthcare professionals; and c) editorials, letters, and reviews.

### 2.4 Screening and selection

Initially, all relevant studies retrieved from each database were imported into Rayyan^®^ [23] to perform a systematic screening. Duplicates were removed and two researchers (FL and SU) independently screened eligibility by title and abstract for all records.

**Fig 1:**
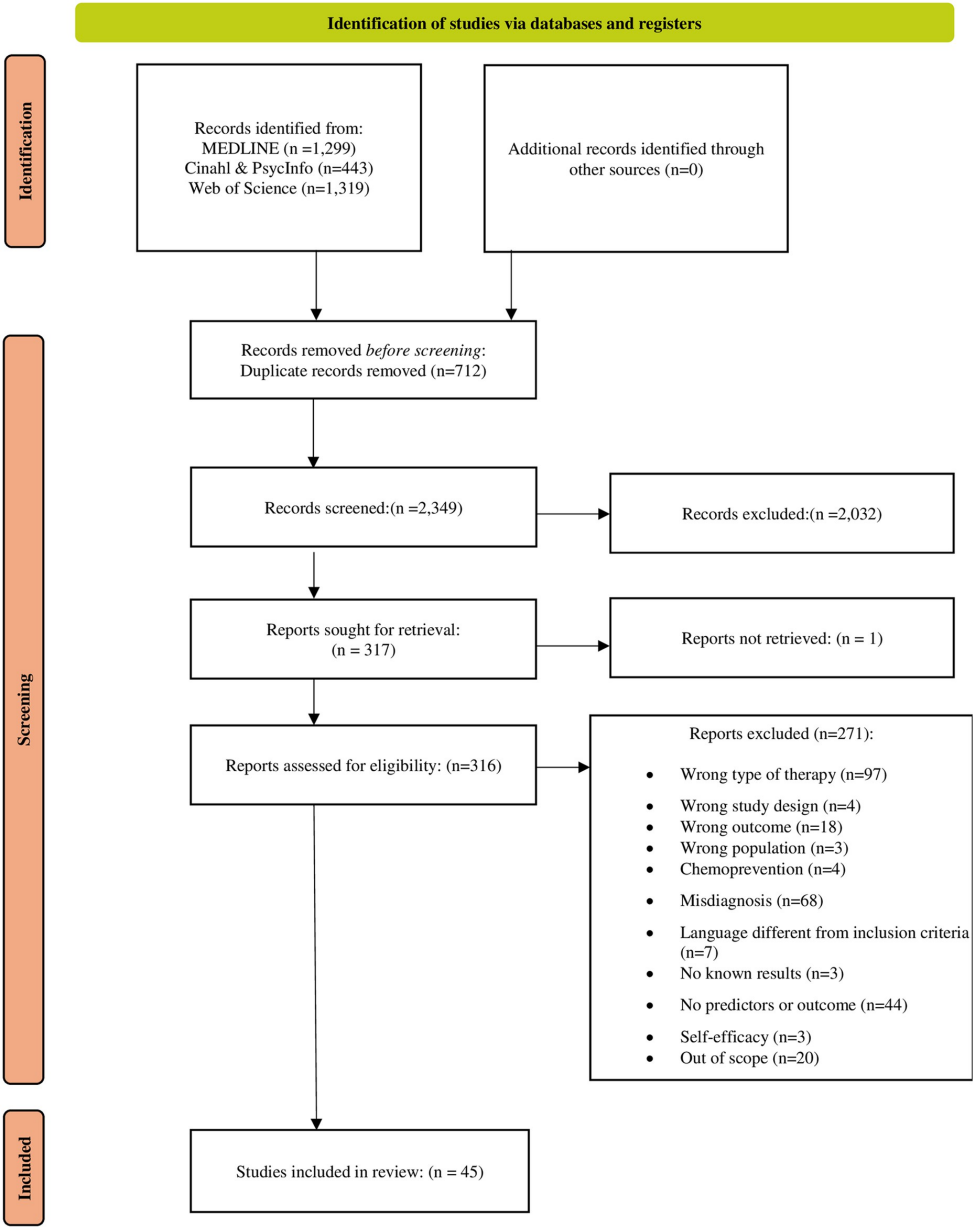
Flow chart—PRISMA.

Using Rayyan^®^ settings, each researcher involved in the screening (FL and SU) was blinded and could not see the choices made by the other researcher. When the selection and data extraction were complete, the blind mode was removed for comparison. Any disagreement was resolved by discussion and consensus. When no consensus was reached, a third researcher expert in OAA (MDN) arbitrarily chose whether the study should be included. Exclusion reasons are provided in Fig 1.

### 2.5 Evaluation of methodological quality

Taking into account eligibility criteria, studies with all designs have been evaluated using the Joanna Briggs Institute [24] critical evaluation tool.This tool is intended to be used as a checklist to assess the methodological quality of a study and to determine the extent to which a study has addressed the possibility of bias in its design, conduct, and analysis [24].

The methodological evaluation allowed one to classify the articles on the basis of a percentage score that was assigned after the research group evaluation. This score was calculated by determining, in percentage, how many items were present in the total of those provided by the JBI checklist. On the basis of the methodological evaluation, a percentage score was assigned to each article and the articles were grouped as follows: studies of low quality (score = 0%-45%), moderate quality (score = 54%-75%), high quality (score = 82%-91%), very high quality (score = 100%).

### 2.6 Data extraction

One researcher (SU) performed the extraction of all relevant data in a standardised form, another checked (FL) the accuracy of these findings. A spreadsheet was used to perform the data extraction.

Any disagreement was resolved by discussion and consensus. When consensus was not achieved, a third member of the research team (MDN) was involved to solve doubts.

Data extracted from the included studies were related to the following fields: author and year of publication, study design, anatomical location of cancer, type of oral anticancer agent, self-care subdimensions, self-care predictors, geographical location and sample size. Odds ratios (OR), hazard ratios (HR), risk ratios (RR), beta coefficient regressions, mean differences with confidence intervals (CI) and p-values were extracted from the different studies and considered as measurements of the effect produced by the results of the studies.

### 2.7 Data synthesis

A narrative (descriptive) synthesis was conducted. To obtain a more comprehensive and comprehensible reading of the predictors extracted from the selected articles, an aggregation of them has been carried out, using, as a guide, the dimensions of self-care predictors provided by "The Situation-Specific Theory of Heart Failure Self-Care" [18]. Specifically, we synthetised the predictors referred to the adult diagnosed with cancer in the “Person” theme, the predictors referred to the physical and emotional consequences of the cancer diagnosis in the “Problem” theme and the predictors referred to the setting in which the person is dealing with the diagnosis of cancer in the “Environment” theme.

## 3. Results

### 3.1 Study selection

As shown in the PRISMA 2020 flow diagram [20] (Fig 1), 3,061 records were retrieved from the database search. After the duplicates were removed, 2,349 records were screened for relevance by title and abstract. Among the 316 full texts examined, 45 studies were included and 271 were excluded.

### 3.2 Study characteristics

We included 45 studies that reported various predictors of adherence that fall in self-care maintenance behaviours in cancer patients using OAA. A summary of the study characteristics is shown in **Table S1B** in S1 Appendix, and a summary of the study design is reported in Table 1.

**Table 1:** Study design of the inclusion study.

Study Design	Authors
Cross-sectional	[25–35]
Quasi-experimental	[36]
RCT	[37, 38]
Cohort	[39–57]
Longitudinal	[58, 59]
Prospective	[60, 61]
Retrospective	[62–65]
Observational	[66–69]

Two studies [27, 29] considered a sample with various cancer diagnoses, including breast, colon, stomach, brain, rectum, and pancreatic cancer. Three studies [33, 39, 61] considered a sample consisting of patients with breast or colorectal diagnosis. The remaining studies examined a population of patients with a single cancer condition [25, 26, 28, 30–32, 34–38, 40–60, 62–69]. In particular, breast cancer was the most prevalent (n = 32) [25, 26, 28, 31, 32, 34–36, 40–47, 49–58, 60, 62, 64, 65, 67, 69], while the less represented population was colorectal cancer (n = 1) [48], lung cancer (n = 1) [59], rectal cancer (n = 1) [66], prostate cancer (n = 2) [38, 68], and metastatic renal cell cancer (n = 2) [37, 63]. Two studies [27, 29] considered a sample with various cancer diagnosis, including breast, colon, stomach, brain, rectum and pancreas cancer.

### 3.3 Methodological quality

The results of the JBI’s critical appraisal tools are presented in Tables from S1C to S1F in S1 Appendix. Four studies with a low-quality score (score = 37.5%-45%), 12 as moderate quality (score = 54%-75%), 15 as high quality (score = 82%-91%), and 14 as very high quality (score = 100%).

### 3.4 Oral anticancer agent therapy

Most studies (24) considered patients with aromatase inhibitors (AI) and tamoxifen [25, 26, 28, 31, 32, 34, 35, 40–43, 47, 49, 52, 54–57, 60, 62, 64, 65, 67, 69], two studies considered selective estrogen receptor modulators (SERMs) or AIs [44, 45], four studies considered only tamoxifen [46, 51, 53, 58], two study considered only AIs [36, 50], four studies considered only capecitabine [27, 33, 39, 66] and all other studies considered more types of OAA [29, 30, 37, 38, 48, 59, 61, 63, 68] (Table S1B in S1 Appendix).

### 3.5 Factors associated with self-care

Of the 45 articles included after extraction of full text data, all included studies reported the dimension of self-care maintenance, specifically reporting adherence. No eligible articles addressed self-care management or self-care monitoring dimensions.

### 3.6 Predictors of self-care maintenance

**Fig 2:**
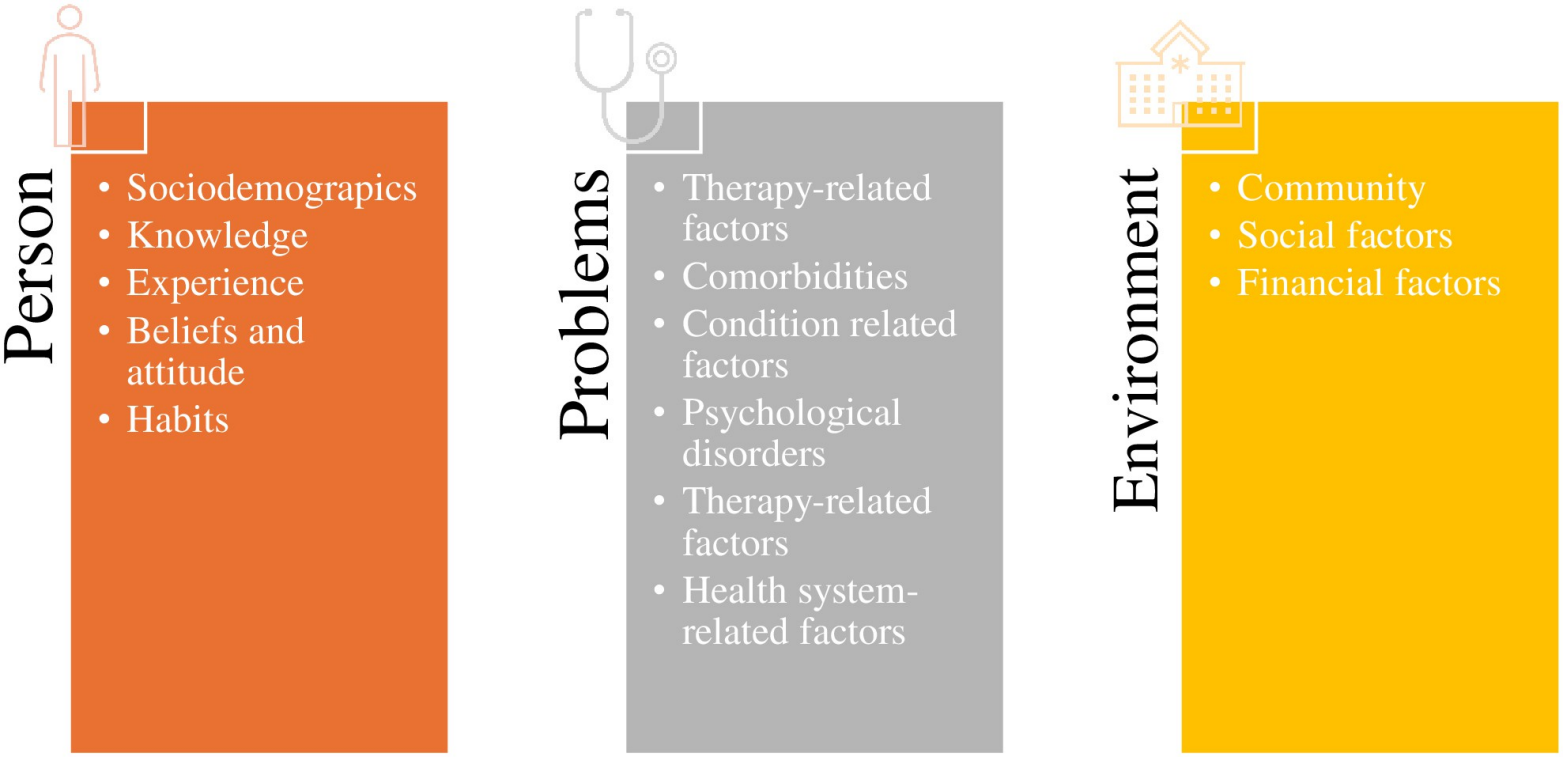
Self-care predictors organised in the three factors influencing self-care.

We identified 46 predictors organised in 14 categories and in turn placed the latest update of self-care theory into three themes accordingly [18] (Fig 2).

Additionally, to systematically classify the predictors and represent the direction of each predictor’s association with adherence, each theme was divided into categories. Each category was then associated with the respective predictors, and their association with adherence to OAAs was described (Table S1G in S1 Appendix).

### 3.7. Person-related predictors

The theme of person-related predictors of self-care relates to the socio-demographic characteristics and experiences of patients diagnosed with cancer under OAA treatment.

#### 3.7.1. Sociodemographic characteristics

Twenty-nine studies [25–27, 31, 32, 35, 36, 41–46, 48, 49, 53, 54, 57–64, 66–69] investigated the association of age with adherence. Eleven studies found that age was significantly associated with adherence. Six studies [44, 45, 53, 57, 67, 69] found that adherence also increased as age increased, particularly in patients older than 70. In contrast, five studies [35, 54, 63, 64, 68] stated that older age was associated with lower adherence rates. Dinan et al. and Pilon et al. reported that being older than 75 years was correlated with lower adherence and there was greater adherence in the age group 55–70 [63, 68], and Keim-Malpass et al. reported that compared to women aged 70 to 75 years, those 85 years or older had a 55% lower likelihood of adhering to adjuvant endocrine therapy [54]. The other 18 studies did not find statistical significance between age and adherence.

Eight studies [27, 33, 48, 49, 57, 59, 63, 66] examined the association between sex and adherence. Only two studies reported statistical significance but with opposite results; Zahrina et al. reported that female sex was significantly associated with greater adherence [33], instead Dinan et al. reported that men were more likely than women to adhere to OAA [63].

Of seven studies [27, 31, 41, 53, 60, 63, 69] that investigated the association between having a partner and adherence, only one study found that there was a significant association between the presence of a partner and higher adherence to treatment [31].

Discordant results were found with respect to ethnicity as included studies compared different ethnicities; Asian patients had 1.2% higher adherence than white patients, and "other" races saw a 3.9% reduction [44]. Haskins et al. [45] reported that being white compared to other ethnicities increased adherence by 0.4%, while being Hispanic or non-Hispanic does not influence adherence. Asian-non-Hispanic and white-Hispanic patients were less likely to adhere than white/non-Hispanic patients [62]. Hu et al. reported that compared to white individuals, being black was associated with 6.5% higher adherence; otherwise, in the study by Pilon et al. black ethnicity was associated with poorer adherence [68]. Compared to white British women, women from other ethnic groups had a much higher risk of nonadherence [67]. Murphy et al. reported that ethnicity was significantly associated with nonadherence [37].

One study found that the place of residence was significantly associated with medication adherence. Patients in the western United States were less likely to adhere to endocrine therapy than those in the Northeast [56].

Employment status was associated with adherence only in two studies. Bourmaud et al. found that retirement was associated with a higher adhesion to OAA [39] but Tinari et al. found that compared to retired people, housewives had a 1.92% higher nonadherence rate [69].

Another factor that emerged was the level of education. In fact, two studies [31, 39] reported that patients with a lower level of education were associated with a higher level of adherence.

#### 3.7.2 Knowledge

Bright et al. [40] reported that any barrier (e.g., difficulty taking the therapy or motivation to take it) to OAA was significantly associated with nonadherence, but reporting the use of any strategy to increase motivation was not significant.

The study by Cakmak & Uncu [29] concluded that there is a positive and strong correlation between health literacy and adherence to OAA. In the experimental study by Yang et al. 2021, the patients of the intervention group, who received an informative guide to implement health literacy about prostate cancer and the side effects of OAA, had a higher adherence rate than the control group [38]. Another study confirmed the association between a lower understanding of the disease and nonadherence [43].

#### 3.7.3 Experience

Adherence was also negatively influenced by previous experience and use of intravenous chemotherapy before starting treatment with OAA [59, 68]. Pilon et al. reported that patients treated with chemotherapy at baseline were 27% less likely to adhere compared to those with no chemotherapy treatment [68]. However, Blanchette et al. reported that prior adjuvant chemotherapy use was associated with higher adherence [64]. Furthermore, as years of OAA use increased, adherence increased [44, 45].

Several studies [26, 27, 33, 34, 40, 60] stated that having a good understanding of treatment, its efficacy, knowledge of risks or benefits, global satisfaction and beliefs about medicine was associated with greater adherence to OAAs. The perception of the need for treatment increased adherence to tamoxifen, AI, and capecitabine. Higher adherence to OAA was reported when patients had positive thoughts about treatment.

The decreased sense of priority for medication was significantly related to medication [30]. The diminished sense of priority for medication was significantly related to medication nonadherence [30].

#### 3.7.4 Habits

Regarding habits, two studies by the same author [44, 45] reported that OAA use was significantly associated with nonadherence.

### 3.8 Problem-related predictors

Forty-five articles explored the association between problem-related predictors and OAA adherence. Problem-related predictors referred to the physical and emotional consequences of the cancer (diagnosis, treatment, or characteristics) on the person. These predictors comprise: comorbidities, including psychological disorders with their therapy and health system-related factors; condition-related factors (for example, stage of the tumour at diagnosis, metastatic state, positive lymph nodes); and therapy-related factors (which include side effects and type of therapy).

#### 3.8.1 Comorbidities

Thirteen out of 45 studies evaluated the presence of comorbidities as a possible predictor of adherence to OAA. Among these, six studies revealed a non-significant association between them [36, 42, 49, 59, 60, 63]. Ali et al. stated that the presence of comorbidities is an independent predictor of adherence in a multiple logistic regression analysis [25] and Corter et al. found that the absence of comorbid conditions was the strongest predictor of actual non-adherence [43]. Furthermore, three studies reported that the increase in the number of comorbidities was associated with an increase in the likelihood of adherence [53, 56, 64].

#### 3.8.2 Psychological disorders

Ten studies out of 45 studies evaluated associations between psychological disorders and OAAs adherence; studies reported that lower adherence was predicted by anxiety and depression [45, 58, 66], anxiety alone [44, 45, 58, 60, 66] and other mental illnesses [45, 58].

#### 3.8.3 Condition related factors

Some characteristics of the tumour at diagnosis were found to be closely related to patient adherence to OAA.

Ten studies evaluated the stage of the tumour at diagnosis as a predictor of adherence to OAA [27, 32, 41, 45, 46, 49, 53, 60, 62, 66]. Three of them reported that the likelihood of adherence to an OAA regimen was reduced as the disease stage increased [32, 41, 45]. The other seven studies did not find statistically significant associations between tumour stage and adherence to OAA [27, 46, 49, 53, 60, 62, 66].

Two studies evaluated the association between positive lymph nodes and adherence to OAAs, reporting no significant results [27, 49].

Two studies considered the ’hormone / progesterone receptor’ in relation to adherence to OAA [49, 56]; Yuan et al. results showed that women with breast cancer and a positive progesterone receptor adhered more to therapy [56], while Valachis et al. found no significant associations between these two variables [49].

Only three of the selected studies investigated whether there was an association between metastatic cancer and OAA adherence with no statistically significant results [48, 49, 68].

Five studies investigated the association between tumour size and adherence [27, 46, 49, 56, 66]; only one of them showed that a tumour size of more than 50 millimetres versus a size of less than 10 mm was significantly associated with increased adherence [56].

Two studies found that ECOG performance status ≥ 1 was associated with increased nonadherence [48, 58], while one study found no significant results [61].

Three studies evaluated recent cancer diagnosis in association with OAA adherence; Haskins et al. found that people who had a more recent cancer diagnosis had higher adherence rates (+8.4% 2013 vs 2007), and with each consecutive year of use, adherence improved [44]. The other two studies did not report significant results [27, 49].

Three studies evaluated associations between complete therapy and adherence; According to Karavites et al. patients who received complete local therapy (mastectomy and radiation therapy) were more adherent than those who did not [46], in contrast to two studies that did not obtain significant results [49, 53].

#### 3.8.4 Other therapies

A total of 14 studies considered taking other therapies in addition to OAA as possible predictors of adherence. Eight of them reported significant associations with adherence to OAA for the following variables: use of selective serotonin reuptake inhibitors (SSRI) [49], polypharmacotherapy [35, 57] prior medication assumption (e.g., analgesics/antipyretics, opiate agonists, cardiac beta blockers, gastrointestinal, etc.) [45, 55] previous regimen of four or more medications [48] and have performed radiation therapy [46, 54].

Valachis et al. found that people with overlapping therapy period < 50% (OAA and SSRI) had a lower adherence to OAA compared to people with overlapping period > 50% [49]. According to Haskins et al. patients with more medications had higher daily adherence [45]; for Neugut et al. people who had assumed prior medications for chronic conditions were more likely to not adhere to OAA [55]. Furthermore, in Sugita et al., having four or more medications regimen was significantly associated with nonadherence to OAA [48]. For Yan et al., women taking Tamoxifen / Toremifene and AI had decreased adherence [57]. Karavites et al. instead found a significant association between previous radiation therapy and the likelihood of adherence [46].

#### 3.8.5 Therapy-related factors

A total of 14 studies investigated the association between side effects (therapy-related factor) and adherence [26, 27, 30–34, 37, 40, 48, 51, 58, 60, 61]. Among these, 12 revealed a statistically significant association between side effects and nonadherence or between side effects and having a lower probability of adherence to OAA [26, 30–34, 37, 40, 51, 58, 60, 61]. Only two articles reported a nonsignificant statistical association between side effects of OAAs and adherence [27, 48]. Furthermore, the avoidance of the occurrence of side effects was found to be related to nonadherence to OAA [25].

Among the side effects, studies reported nausea, vomiting and diarrhoea [30, 33, 46, 48] musculoskeletal pain [30, 46, 48, 58], malaise [46, 58], neutropenia [48], reduction of concentration [31], gynaecological symptoms such as vaginal dryness [58], hand-foot syndrome [61], skin rash, desquamation, and acne [37].

Ten articles evaluated whether the type of anticancer therapy could be a predictor of adherence / nonadherence to OAA [25, 27, 31, 32, 36, 42, 60–63].

In eight of 12 studies, the type of OAA or the prescribed therapy scheme was not significantly associated with the probability of adherence to the medication [25, 27, 31, 32, 36, 42, 60, 61].

Four articles reported a statistically significant association between the type of anticancer therapy and the likelihood of adherence [37, 62–64]. In Hwang et al. patients who assumed AIs were more likely to adhere compared to patients who assumed Tamoxifen [62]. Dinan et al. demonstrated that compared to sunitinib patients, adherence was 37% lower in patients using Pazopanib and 47% lower in patients using Sorafenib.

Regarding the therapy scheme (e.g., taking the therapy depending on the therapy schedule), Hirao et al. reported that the timing (in this study, taking OAA every eight hours) was a determinant of non-adherence [30]. Only three studies evaluated the association between the duration of treatment and the adherence to OAA: among these, two studies reported a nonsignificant association [36, 42] and only one study reported a significant association between duration of the therapy and adherence; specifically people taking OAAs from three to five years had a lower adherence compared to patients taking OAAs for two years or less [62]. Likewise, three studies analysed the association between time since diagnosis and adherence. Two studies reported a non-significant association [27, 53] and one study a significant increase in adherence for increasing years of OAA intake since diagnosis [45]. Switching to another OAA was not a predictor of adherence in two studies [60, 62] but it was associated with nonadherence in another one [69].

#### 3.8.6 Health system-related factors

Four studies considered factors related to the health system as potential predictors of OAAs adherence; three of these evaluated the personalised care plan and one the specialistic care as predictors of adherence. Studies that focused on a personalised care plan reported that patients who had multiple visits with their oncologists, with the possibility of receiving more information about their problem and treatment characteristics, were more likely to adhere to the OAAs regimen [28, 45, 64]. A study found a nonsignificant association between oncology visits before anticancer assumption and adherence to OAA adherence [46].

### 3.9 Environment-related predictors

The environment refers to the context in which the person is coping with the path of cancer diagnosis.

#### 3.9.1 Community factors

Haskins et al. reported that living in an urban area, compared to the rural area, was associated with less adherence [45] but Oke et al. reported that residents in metropolitan or less urban areas had a higher probability of adherence [53]. Furthermore, one study reported that the probability of early discontinuation increased as neighbourhood deprivation increased [63].

#### 3.9.2 Social factors

Only one study explored the social support factor and reported that patients with family obligations were more likely to be non-adherent [39].

#### 3.9.3 Financial factors

Several studies [36, 41, 42, 45–47, 50, 53, 57–59, 63, 64, 68] reported an association between economic factors and adherence to OAA.

Patients with health insurance had a greater adherence to tamoxifen and AIs [46]. Pilon et al. reported that compared to patients with commercial insurance, Medicare patients were 23% more likely to adhere [68]. Patients who received the subsidy had significantly higher adherence throughout the studies considered [36, 47, 50, 59]. Ma et al. reported that patients who received a low-income subsidy were 11.4% more likely to adhere to AIs [47].

Three studies have analysed the impact of the introduction of generic AIs on adherence [36, 47, 50]. The introduction of a generic AI (anastrozole) showed a quarterly increase in the probability of adherence. After the introduction of the other two generics, letrozole and exemestane, there was a further 0.8% quarterly increase in the probability of adherence among beneficiaries of non-low-income subsidies [47]. The authors did not find a statistically significant difference in the probability of adherence before and after the introduction of generic aromatase inhibitors among LIS beneficiaries [47]. Neuner’s study also showed that adherence to AIs among LIS beneficiaries did not change after the introduction of generic equivalents. In contrast, among non-LIS beneficiaries, there was a marked reduction in the rate of decline in anastrozole adherence with the introduction of generic anastrozole [36]. On the contrary, Winn et al. did not find a change in adherence after the introduction of generic AIs among patients who received and did not receive a subsidy [50].

A reduction in the monthly copayment amount was significantly associated with a higher likelihood of adherence [42].

Women who had prescribed medicine coverage were more likely to initiate and continue tamoxifen or AIs to women without prescription drug coverage. In addition, annual household income was associated with the beginning and continuation of hormonal therapy. Women with the lowest income were less likely to continue hormonal therapy compared to women with an annual household income greater than $70,000 [41].

Higher out-of-pocket costs were significantly associated with a higher risk of nonadherence [42, 45, 59, 63]. In detail, Hess et al. estimated that each $100 increase in out-of-pocket costs was associated with a 6.0% decrease in adherence. Patients who paid less than the median out of pocket had a 22% higher likelihood of adhering to treatment than those who paid more than the median [68].

## 4. Discussion

This systematic review provides an overview of the available studies that describe predictors that may be related to self-care behaviour in patients taking OAA. Specifically, we focused on self-care maintenance, monitoring, and management behaviours, according to the theory of Riegel [18]. Our review shows that the only dimension of self-care studied in the literature was self-care maintenance and specifically all studies referred to medication adherence. A better understanding of the predictive and modifiable factors that influence self-care of patients on OAAs can improve cancer outcomes, including risk of recurrence, disease-free survival, and quality of life.

### 4.1 Person-related predictors

This systematic review identified several factors that predict high or low adherence to OAA. The results of the included studies suggest that age is a strong predictor, but without a definitive direction for adherence, and therefore it is unclear whether adherence may depend on a lower or higher age group, and therefore no firm conclusions can be drawn. It would be expected that older people would be more likely to be non-adherent for various reasons, such as cognitive deficits, comorbidities, polypharmacy, financial burdens, or that they may perceive less benefit from OAA and have more concerns about potential side effects or toxicity [70]. However, our study found that adherence can increase with age. Therefore, it can be argued that young cancer patients should be considered a vulnerable group of patients. This may be due to greater psychosocial distress, in particular greater difficulty in coping with the disease. To support this, a review aimed at exploring the factors that cause nonadherence to medical therapy reported that young working women are more likely to be poorly adherent due to work-family balance [71].

Patient health literacy is crucial because it can provide an understanding of the many treatment options and their adverse effects, as well as facilitate conversations with healthcare professionals [72]. In several studies there is a significant relationship between health literacy and adherence [29, 38, 40, 43]. Similarly, health literacy can influence patient beliefs about medication (such as perceived need, perceived concerns, or perceived risks and benefits of treatment), which have been shown to significantly influence medication adherence. Furthermore, patients who are more satisfied with the information provided have been found to be more adherent.

### 4.2 Problem-related predictors

Among the predictors reported, the ones related to therapy were the most representative. Most studies reported that patients who developed side effects were less adherent than those who did not [26, 30, 31, 34, 40, 51, 60, 61]. The reasons for the development of side effects in people taking OAA can vary and can be worsened by polypharmacotherapy.

Cancer patients often take other medications for comorbidities, nutritional supplements, or other complementary therapies that can often generate interactions [73]. Sometimes, they are affected by chronic renal failure due to previous chemotherapy intake, so they develop side effects of renal toxicities. Probably the reason why side effects lead to nonadherence to OAA is the negative impact generated on the individual’s quality of life [74, 75].

Interestingly, a study [60] reported that only having side effects (without considering their amount) is associated with nonadherence to OAAs. The impossibility to define an association between the number of side effects and adherence could be due, on the one hand, to the way the results of this association are influenced by how the patient operationalises the concept of ‘side effect’ [60]. Patients often tend to report fewer side effects because they fear that the oncologist could reduce the dose of the drug, making the treatment less effective [37, 76]. On the other hand, for the oncology team, it is more difficult to adequately monitor when side effects occur considering that OAA is primarily managed at home, relying solely on what the patient (or caregiver) reports. Therefore, it is very important to provide patients with all the information on self-assessment and self- monitoring of side effects they could develop at home.

Nurses can play a crucial role in avoiding situations that lead to side effects and, consequently, to negative patient outcomes. They can detect the presence of side effects using, for example, telemonitoring systems [77] instead of an assessment during oncology visits, and they can implement person-centred pathways that reduce side effects impairment in patient quality of life [78, 79]. Furthermore, nurses are an important reference for the patient on OAA, as they collect requests for information on the treatment scheme and promote the development of patient self-efficacy [80]. The latter has been shown to be a mediator for the development of self-care behaviours that allow the patient to recognise and manage side effects [81].

The type of OAA was another predictor extensively explored, as 10 articles investigated its association with adherence. For this predictor, the results obtained are conflicting because the authors of the included studies have reported how taking a drug or a combination of them may be associated with greater or less adherence [25, 27, 31, 32, 36, 42, 60–63]. This result highlights how the type of OAA, although it can be considered a predictor of therapeutic adherence, must also be evaluated considering the specific characteristics of the patient and his diagnosis, such as cancer stage. In fact, many studies revealed that patients put more emphasis on taking OAA if they are diagnosed with metastatic cancer than non-metastatic cancer [82].

Another important predictor of adherence, resulting from this review, was the presence of comorbidities. Most of the included articles stated that patients with comorbidities had lower adherence, if the comorbidity was anxiety or a mental disorder [45, 58, 66]. This may be due to the impairment and frailty condition that a mental disorder induces in people [75, 83] even if there are few studies on this topic due to the impossibility of enrolling patients with mental illness in cohort studies. Furthermore, the lower adherence caused by the presence of comorbidities could be related to the number of medications a patient with comorbidities must take in combination with OAA.

### 4.3 Environment-related predictors

Socioeconomic status has been found to be associated with access to care. Cost factors may influence adherence to treatment. We found that insurance status can be a predictor of nonadherence to OAA [46, 57]. In countries with insurance-based healthcare systems, patients without insurance may struggle with extra monthly expenses and therefore must prioritise other needs. Therefore, it is possible that patients who have out-of-pocket expenses for their care are more likely to abandon therapy in the event of side effects. In two studies [36, 47] the use of a generic equivalent medicine significantly increased patient adherence because it reduced and/or eliminated high out-of-pocket costs for financially vulnerable patients. As a result, healthcare policies can improve equity in the use of healthcare services, reducing disparities and negative outcomes (e.g., mortality, low quality of life).

## 5. Limitations

This systematic review has some limitations. First, the search process was performed using only three databases (PubMed, Cinahl & PsycInfo and Web of Science), and the grey literature was not searched. Second, the concept of self-care in the literature was still little studied, and only the concept of adherence, considered as an element of self-care maintenance, was investigated. Moreover, at the time the search strategy was built, the protocol also included the assessment of self-care outcomes. However, due to the large number of articles that emerged on the predictors of self-care, only the results on the predictors of self-care were reported in this article in order to give an in-depth view of these results.

## 6. Conclusions

In general, most of the included studies focused only on adherence. More research is needed to deepen what the predictors of self-care are in all its dimensions. The results of this review converge to define which are the main predictors that increase adherence to OAA as part of self-care maintenance behaviours: a better relationship between the patient and the health providers, having social support, not addiction to alcohol or drugs, having health insurance or financial support, being however side effects one of the most reported factors that negatively affect adherence.

The debate is open about important factors such as age, gender, education, comorbidities, polypharmacy therapy. It is important for the oncology nurse to know the factors that affect self-care to prevent negative treatment outcomes (e.g., side effects recurrence, rehospitalizations, access to the emergency room, and reduction of quality of life).

## Supporting information

S1 Appendix Appendix includes: **Table S1A:** Search Strategy. **Table S1B**. Characteristics of studies included. **Table S1C**. Methodological quality assessment of studies (cross-sectional and observational). **Table S1D**. Methodological quality assessment of studies (quasi-experimental). **Table S1E**. Assessment of methodological quality, with the jbi checklist, for cohort studies (numeric). **Table S1F**. Quality assessment with new version of JBI check list for RCT studies. **Table S1G**. Results from included studies.
(DOCX)

## Abbreviations

AIs: Aromatase Inhibitors
JBI: Johanna Briggs Institute
LIS: Low Income Subsidy
OAAs: Oral Anticancer Agents
PRISMA: Preferred Reporting Items for Systematic Reviews and Meta-Analyses
SERMS: Selective Estrogen Receptor Modulator

## References

[1] Neuss MN, Polovich M, McNiff K, Esper P, Gilmore TR, LeFebvre KB, et al. 2013 Updated American Society of Clinical Oncology/Oncology Nursing Society Chemotherapy Administration Safety Standards Including Standards for the Safe Administration and Management of Oral Chemotherapy. 2013;9(2S):5s–13s. doi: 10.1200/jop.2013.000874 . 23914148 PMC3731124

[2] Leong C, Czaykowski P, Geirnaert M, Katz A, Dragan R, Yogendran M, et al. Outpatient oral anticancer agent utilization and costs in Manitoba from 2003 to 2016: a population-based study. Canadian Journal of Public Health. 2021;112(3):530–40. doi: 10.17269/s41997-020-00464-6 33471346 PMC8076393

[3] Eek D, Krohe M, Mazar I, Horsfield A, Pompilus F, Friebe R, et al. Patient-reported preferences for oral versus intravenous administration for the treatment of cancer: a review of the literature. Patient Prefer Adherence. 2016;10:1609–21. Epub 2016/09/08. doi: 10.2147/PPA.S106629 . 27601886 PMC5003561

[4] Barillet M, Prevost V, Joly F, Clarisse B. Oral antineoplastic agents: how do we care about adherence? Br J Clin Pharmacol. 2015;80(6):1289–302. Epub 2015/08/11. doi: 10.1111/bcp.12734 . 26255807 PMC4693496

[5] Jacobs JM, Pensak NA, Sporn NJ, MacDonald JJ, Lennes IT, Safren SA, et al. Treatment Satisfaction and Adherence to Oral Chemotherapy in Patients With Cancer. Journal of Oncology Practice. 2017;13(5):e474–e85. doi: 10.1200/JOP.2016.019729 . 28398843

[6] Banna GL, Urzia V, Benanti C, Pitrè A, Lipari H, Di Quattro R, et al. Adherence to abiraterone or enzalutamide in elderly metastatic castration-resistant prostate cancer. Supportive Care in Cancer. 2020;28(10):4687–95. doi: 10.1007/s00520-020-05311-5 31960124

[7] Gebbia V, Bellavia M, Banna GL, Russo P, Ferrau F, Tralongo P, et al. Treatment Monitoring Program for Implementation of Adherence to Second-Line Erlotinib for Advanced Non-Small-Cell Lung Cancer. CLINICAL LUNG CANCER. 2013;14(4):390–8. doi: 10.1016/j.cllc.2012.11.007 23313173

[8] Vachon E, Given B, Given C, Dunn S. Temporary stoppages and burden of treatment in patients with cancer. Oncol Nurs Forum. 2019;46(5):E135–e44. Epub 2019/08/20. doi: 10.1188/19.ONF.E135-E144 . 31424460 PMC7360060

[9] Di Nitto M, Sollazzo F, Biagioli V, Pucciarelli G, Torino F, Alvaro R, et al. Self-care behaviors in patients with cancer treated with oral anticancer agents: a systematic review. Supportive Care in Cancer. 2022;30(10):8465–83. doi: 10.1007/s00520-022-07166-4 35639188

[10] Riegel B, Jaarsma T, Strömberg A. A Middle-Range Theory of Self-Care of Chronic Illness. Advances in Nursing Science. 2012;35(3). doi: 10.1097/ANS.0b013e318261b1ba 22739426

[11] Biagioli V, Drury A, Wells M, Eicher M, Kelly D. Self-care and cancer: Comment on Riegel et al. (2020) ’Characteristics of self-care interventions for patients with a chronic condition: A scoping review’. Int J Nurs Stud. 2021;115:103877. Epub 2021/01/20. doi: 10.1016/j.ijnurstu.2021.103877 . 33465578

[12] Jaarsma T, Hill L, Bayes-Genis A, La Rocca HB, Castiello T, Čelutkienė J, et al. Self-care of heart failure patients: practical management recommendations from the Heart Failure Association of the European Society of Cardiology. Eur J Heart Fail. 2021;23(1):157–74. Epub 2020/09/19. doi: 10.1002/ejhf.2008 . 32945600 PMC8048442

[13] Ausili D, Rossi E, Rebora P, Luciani M, Tonoli L, Ballerini E, et al. Socio-demographic and clinical determinants of self-care in adults with type 2 diabetes: a multicentre observational study. Acta Diabetologica. 2018;55(7):691–702. doi: 10.1007/s00592-018-1135-x 29623431

[14] Jaarsma T, Cameron J, Riegel B, Stromberg A. Factors Related to Self-Care in Heart Failure Patients According to the Middle-Range Theory of Self-Care of Chronic Illness: a Literature Update. Current Heart Failure Reports. 2017;14(2):71–7. doi: 10.1007/s11897-017-0324-1 28213768 PMC5357484

[15] Kamrani A, Foroughan M, Taraghi Z, Yazdani J, Kaldi A-R, Ghanei N, et al. Self care behaviors among elderly with chronic heart failure and related factors. Pakistan journal of biological sciences: PJBS. 2014;17(11):1161–9. doi: 10.3923/pjbs.2014.1161.1169 26027161

[16] Montagna E, Zagami P, Masiero M, Mazzocco K, Pravettoni G, Munzone E. Assessing Predictors of Tamoxifen Nonadherence in Patients with Early Breast Cancer. Patient Prefer Adherence. 2021;15:2051–61. Epub 2021/09/24. doi: 10.2147/PPA.S285768 . 34552323 PMC8450184

[17] Jiang Y, Wickersham KE, Zhang X, Barton DL, Farris KB, Krauss JC, et al. Side Effects, Self-Management Activities, and Adherence to Oral Anticancer Agents. Patient Prefer Adherence. 2019;13:2243–52. Epub 20191231. doi: 10.2147/PPA.S224496 . 32099335 PMC6997414

[18] Riegel B, Dickson VV, Vellone E. The Situation-Specific Theory of Heart Failure Self-care: An Update on the Problem, Person, and Environmental Factors Influencing Heart Failure Self-care. J Cardiovasc Nurs. 2022. Epub 2022/04/29. doi: 10.1097/JCN.0000000000000919 . 35482335 PMC9561231

[19] Higgins JP, Thomas J, Chandler J, Cumpston M, Li T, Page MJ, et al. Cochrane handbook for systematic reviews of interventions: John Wiley & Sons; 2019. 10.1002/14651858.ED000142 PMC10284251 31643080

[20] Page MJ, McKenzie JE, Bossuyt PM, Boutron I, Hoffmann TC, Mulrow CD, et al. The PRISMA 2020 statement: an updated guideline for reporting systematic reviews. 2021;372:n71. doi: 10.1136/bmj.n71%J BMJ. 33782057 PMC8005924

[21] Riegel B, Jaarsma T, Lee CS, Strömberg A. Integrating Symptoms Into the Middle-Range Theory of Self-Care of Chronic Illness. ANS Adv Nurs Sci. 2019;42(3):206–15. Epub 2018/11/27. doi: 10.1097/ANS.0000000000000237 . 30475237 PMC6686959

[22] Riegel B, Dickson VV, Faulkner KM. The Situation-Specific Theory of Heart Failure Self-Care: Revised and Updated. J Cardiovasc Nurs. 2016;31(3):226–35. Epub 2015/03/17. doi: 10.1097/JCN.0000000000000244 . 25774844

[23] Ouzzani M, Hammady H, Fedorowicz Z, Elmagarmid A. Rayyan—a web and mobile app for systematic reviews. Systematic Reviews. 2016;5(1):210. doi: 10.1186/s13643-016-0384-4 27919275 PMC5139140

[24] JBI. JBI Manual for Evidence Synthesis. In: Aromataris E MZ, editor. 2020.

[25] Ali EE, Cheung KL, Lee CP, Leow JL, Yap KY, Chew L. Prevalence and Determinants of Adherence to Oral Adjuvant Endocrine Therapy among Breast Cancer Patients in Singapore. Asia Pac J Oncol Nurs. 2017;4(4):283–9. doi: 10.4103/2347-5625.212864 28966955 PMC5559937

[26] Brett J, Fenlon D, Boulton M, Hulbert-Williams NJ, Walter FM, Donnelly P, et al. Factors associated with intentional and unintentional non-adherence to adjuvant endocrine therapy following breast cancer. EUROPEAN JOURNAL OF CANCER CARE. 2018;27(1). doi: 10.1111/ecc.12601 27901302

[27] Hefner J, Berberich S, Lanvers E, Sanning M, Steimer AK, Kunzmann V. Patient doctor relationship and adherence to capecitabine in outpatients of a German comprehensive cancer center. PATIENT PREFERENCE AND ADHERENCE. 2018;12:1875–87. doi: 10.2147/PPA.S169354 30288028 PMC6159803

[28] Arriola KRJ, Mason TA, Bannon KA, Holmes C, Powell CL, Horne K, et al. Modifiable risk factors for adherence to adjuvant endocrine therapy among breast cancer patients. PATIENT EDUCATION AND COUNSELING. 2014;95(1):98–103. 24492157 10.1016/j.pec.2013.12.019

[29] Cakmak HSG, Uncu D. Relationship between Health Literacy and Medication Adherence of Turkish Cancer Patients Receiving Oral Chemotherapy. ASIA-PACIFIC JOURNAL OF ONCOLOGY NURSING. 2020;7(4):365–9. doi: 10.4103/apjon.apjon_30_20 33062832 PMC7529023

[30] Hirao C, Mikoshiba N, Shibuta T, Yamahana R, Kawakami A, Tateishi R, et al. Adherence to oral chemotherapy medications among gastroenterological cancer patients visiting an outpatient clinic. JAPANESE JOURNAL OF CLINICAL ONCOLOGY. 2017;47(9):786–94. doi: 10.1093/jjco/hyx087 28633481

[31] Iacorossi L, Gambalunga F, Fabi A, Giannarelli D, Facchinetti G, Piredda M, et al. Adherence to hormone therapy in women with breast cancer: a quantitative study. Prof Inferm. 2016;69(4):113–21. doi: 10.7429/pi.2016.692113 27600553

[32] Stahlschmidt R, Ferracini AC, de Souza CM, de Medeiros LM, Juliato CRT, Mazzola PG. Adherence and quality of life in women with breast cancer being treated with oral hormone therapy. Support Care Cancer. 2019;27(10):3799–804. Epub 2019 Feb 6. doi: 10.1007/s00520-019-04671-x 30729297

[33] Zahrina AK, Norsa’adah B, Hassan NB, Norazwany Y, Norhayati I, Roslan MH, et al. Adherence to capecitabine treatment and contributing factors among cancer patients in Malaysia. Asian Pac J Cancer Prev. 2014;15(21):9225–32. doi: 10.7314/apjcp.2014.15.21.9225 25422205

[34] Koni AA, Suwan BA, Nazzal MA, Sleem A, Daifallah A, Allah MH, et al. Adherence to oral anticancer hormonal therapy in breast cancer patients and its relationship with treatment satisfaction: an important insight from a developing country. BMC Womens Health. 2023;23(1):114. Epub 2023/03/22. doi: 10.1186/s12905-023-02276-5 . 36941628 PMC10026465

[35] Pourcelot C, Orillard E, Nallet G, Dirand C, Billion-Rey F, Barbier G, et al. Adjuvant hormonal therapy for early breast cancer: an epidemiologic study of medication adherence. Breast Cancer Res Treat. 2018;169(1):153–62. doi: 10.1007/s10549-018-4676-3 29362956

[36] Neuner JM, Kamaraju S, Charlson JA, Wozniak EM, Smith EC, Biggers A, et al. The Introduction of Generic Aromatase Inhibitors and Treatment Adherence Among Medicare D Enrollees. JNCI-JOURNAL OF THE NATIONAL CANCER INSTITUTE. 2015;107(8). doi: 10.1093/jnci/djv130 25971298 PMC4580559

[37] Murphy CC, Fullington HM, Gerber DE, Bowman IA, Puligandla M, Dutcher JP, et al. Adherence to oral therapies among patients with renal cell carcinoma: Post hoc analysis of the ECOG-ACRIN E2805 trial. CANCER MEDICINE. 10.1002/cam4.4140 PMC8419781 34405965

[38] Yang R, Lu Z, Gu X, Dai B. The Effect of an Information Support Program on Self-Efficacy of Prostate Cancer Patients during Hormonal Therapy. Asia-Pacific Journal of Oncology Nursing. 2021;8(6):639–52. 152948851. Language: English. Entry Date: doi: 10.4103/apjon.apjon-2138 . Revision Date: 20211026. Publication Type: Article. 34790848 PMC8522598

[39] Bourmaud A, Henin E, Tinquaut F, Regnier V, Hamant C, Colomban O, et al. Adherence to oral anticancer chemotherapy: What influences patients’ over or non-adherence? Analysis of the OCTO study through quantitative-qualitative methods. BMC Res Notes. 2015;8:291-. doi: 10.1186/s13104-015-1231-8 26142140 PMC4490730

[40] Bright EE, Petrie KJ, Partridge AH, Stanton AL. Barriers to and facilitative processes of endocrine therapy adherence among women with breast cancer. Breast Cancer Res Treat. 2016;158(2):243–51. doi: 10.1007/s10549-016-3871-3 27342455

[41] Bradley CJ, Dahman B, Jagsi R, Katz S, Hawley S. Prescription drug coverage: implications for hormonal therapy adherence in women diagnosed with breast cancer. BREAST CANCER RESEARCH AND TREATMENT. 2015;154(2):417–22. doi: 10.1007/s10549-015-3630-x 26553168 PMC4653093

[42] Chin AL, Bentley JP, Pollom EL. The impact of state parity laws on copayments for and adherence to oral endocrine therapy for breast cancer. CANCER. 2019;125(3):374–81. doi: 10.1002/cncr.31910 30566762 PMC6340721

[43] Corter AL, Broom R, Porter D, Harvey V, Findlay M. Predicting nonadherence to adjuvant endocrine therapy in women with early stage breast cancer. PSYCHO-ONCOLOGY. 2018;27(9):2096–103. doi: 10.1002/pon.4771 29776011

[44] Haskins CB, McDowell BD, Carnahan RM, Fiedorowicz JG, Wallace RB, Smith BJ, et al. Impact of preexisting mental illness on breast cancer endocrine therapy adherence. Breast Cancer Res Treat. 2019;174(1):197–208. doi: 10.1007/s10549-018-5050-1 30465157 PMC6426454

[45] Haskins CB, Neuner JM, McDowell BD, Carnahan RM, Fiedorowicz JG, Wallace RB, et al. Effects of Previous Medication Regimen Factors and Bipolar and Psychotic Disorders on Breast Cancer Endocrine Therapy Adherence. Clin Breast Cancer. 2020;20(3):e261–e80. doi: 10.1016/j.clbc.2019.09.005 32139273 PMC7103521

[46] Karavites LC, Kane AK, Zaveri S, Xu YF, Helenowski I, Hansen N, et al. Tamoxifen Acceptance and Adherence among Patients with Ductal Carcinoma In Situ (DCIS) Treated in a Multidisciplinary Setting. CANCER PREVENTION RESEARCH. 2017;10(7):389–97. doi: 10.1158/1940-6207.CAPR-17-0029 28559459

[47] Ma SY, Shepard DS, Ritter GA, Martell RE, Thomas CP. The impact of the introduction of generic aromatase inhibitors on adherence to hormonal therapy over the full course of 5-year treatment for breast cancer. CANCER. 2020;126(15):3417–25. doi: 10.1002/cncr.32976 32484941

[48] Sugita K, Kawakami K, Yokokawa T, Sugisaki T, Takiguchi T, Aoyama T, et al. Self-Reported Adherence to Trifluridine and Tipiracil Hydrochloride for Metastatic Colorectal Cancer: A Retrospective Cohort Study. ONCOLOGY. 2016;91(4):224–30. doi: 10.1159/000448717 27513940

[49] Valachis A, Garmo H, Weinman J, Fredriksson I, Ahlgren J, Sund M, et al. Effect of selective serotonin reuptake inhibitors use on endocrine therapy adherence and breast cancer mortality: a population-based study. Breast Cancer Res Treat. 2016;159(2):293–303. doi: 10.1007/s10549-016-3928-3 27492739 PMC5012147

[50] Winn AN, Fergestrom NM, Neuner JM. Using Group-based Trajectory Models and Propensity Score Weighting to Detect Heterogeneous Treatment Effects The Case Study of Generic Hormonal Therapy for Women With Breast Cancer. MEDICAL CARE. 2019;57(1):85–93. doi: 10.1097/MLR.0000000000001019 30489546 PMC6291347

[51] Zeidan B, Anderson K, Peiris L, Rainsbury D, Laws S. The impact of tamoxifen brand switch on side effects and patient compliance in hormone receptor positive breast cancer patients. Breast. 2016;29:62–7. doi: 10.1016/j.breast.2016.07.001 27428472

[52] Rassy E, Bardet A, Bougacha O, Gantzer L, Lekens B, Delaloge S, et al. Association of Adherence to Endocrine Therapy Among Patients With Breast Cancer and Potential Drug-Drug Interactions. JAMA NETWORK OPEN. 2022;5(12). doi: 10.1001/jamanetworkopen.2022.44849 36459136 PMC9719053

[53] Oke O, Niu J, Chavez-MacGregor M, Zhao H, Giordano SH. Adjuvant tamoxifen adherence in men with early-stage breast cancer. Cancer. 2022;128(1):59–64. doi: 10.1002/cncr.33899 34597415 PMC11927788

[54] Keim-Malpass J, Anderson RT, Balkrishnan R, Desai RP, Showalter SL. Evaluating the Long-Term Impact of a Cooperative Group Trial on Radiation Use and Adjuvant Endocrine Therapy Adherence Among Older Women. ANNALS OF SURGICAL ONCOLOGY. 2020;27(9):3458–65. doi: 10.1245/s10434-020-08430-9 32270421 PMC12038951

[55] Neugut AI, Zhong X, Wright JD, Accordino M, Yang J, Hershman DL. Nonadherence to Medications for Chronic Conditions and Nonadherence to Adjuvant Hormonal Therapy in Women With Breast Cancer. JAMA Oncol. 2016;2(10):1326–32. doi: 10.1001/jamaoncol.2016.1291 27281650

[56] Yuan C, Xie Z, Bian J, Huo J, Daily K. Outcomes of primary endocrine therapy in elderly women with stage I-III breast cancer: a SEER database analysis. Breast Cancer Res Treat. 2020;180(3):819–27. doi: 10.1007/s10549-020-05591-9 32172303

[57] Yan YD, Fu J, Gu ZC, Lu JS, Su YJ, Lin HW. Adherence to endocrine therapy in patients with hormone receptor-positive early-stage breast cancer: a retrospective study. Int J Clin Pharm. 2023;45(1):184–90. Epub 2022/11/17. doi: 10.1007/s11096-022-01450-3 . 36383338

[58] Bender CM, Gentry AL, Brufsky AM, Casillo FE, Cohen SM, Dailey MM, et al. Influence of patient and treatment factors on adherence to adjuvant endocrine therapy in breast cancer. Oncol Nurs Forum. 2014;41(3):274–85. doi: 10.1188/14.ONF.274-285 24769592 PMC4090095

[59] Hess LM, Louder A, Winfree K, Zhu YE, Oton AB, Nair R. Factors Associated with Adherence to and Treatment Duration of Erlotinib Among Patients with Non-Small Cell Lung Cancer. J Manag Care Spec Pharm. 2017;23(6):643–52. doi: 10.18553/jmcp.2017.16389 28530522 PMC10397790

[60] Pan YQ, Heisig SR, von Blanckenburg P, Albert US, Hadji P, Rief W, et al. Facilitating adherence to endocrine therapy in breast cancer: stability and predictive power of treatment expectations in a 2-year prospective study. BREAST CANCER RESEARCH AND TREATMENT. 2018;168(3):667–77. 29330625 10.1007/s10549-017-4637-2 PMC5842254

[61] Vacher L, Thivat E, Poirier C, Mouret-Reynier MA, Chollet P, Devaud H, et al. Improvement in adherence to Capecitabine and Lapatinib by way of a therapeutic education program. SUPPORTIVE CARE IN CANCER. 2020;28(7):3313–22. doi: 10.1007/s00520-019-05144-x 31758323

[62] Hwang GS, Paranjpe R, Opsomer C, Lu K, Abajue U, Abughosh S, et al. Oral Endocrine Therapy Agent, Race/Ethnicity, and Time on Therapy Predict Adherence in Breast Cancer Patients in a Large Academic Institution. CLINICAL BREAST CANCER. 2020;20(6):520–6. doi: 10.1016/j.clbc.2020.06.004 32669209

[63] Dinan MA, Wilson LE, Greiner MA, Spees LP, Pritchard JE, Zhang T, et al. Oral Anticancer Agent (OAA) Adherence and Survival in Elderly Patients With Metastatic Renal Cell Carcinoma (mRCC). Urology. 2022;168:129–36. Epub 2022/07/26. doi: 10.1016/j.urology.2022.07.012 . 35878815 PMC9588695

[64] Blanchette PS, Lam M, Richard L, Allen B, Shariff SZ, Vandenberg T, et al. Factors associated with endocrine therapy adherence among post-menopausal women treated for early-stage breast cancer in Ontario, Canada. Breast Cancer Res Treat. 2020;179(1):217–27. doi: 10.1007/s10549-019-05430-6 31571072

[65] Hu X, Walker MS, Stepanski E, Kaplan CM, Martin MY, Vidal GA, et al. Racial differences in patient-reported symptoms and adherence to adjuvant endocrine therapy among women with early-stage, hormone receptor—positive breast cancer. JAMA Network Open. 2022;5(8):e2225485–e. doi: 10.1001/jamanetworkopen.2022.25485 35947386 PMC9366541

[66] Font R, Espinas JA, Layos L, Villacampa MM, Capdevila J, Tobena M, et al. Adherence to capecitabine in preoperative treatment of stage II and III rectal cancer: do we need to worry? ANNALS OF ONCOLOGY. 2017;28(4):831–5. doi: 10.1093/annonc/mdx006 28327898

[67] McGuinness S, Hughes L, Moss-Morris R, Hunter M, Norton S, Moon Z. Adherence to adjuvant endocrine therapy among White British and ethnic minority breast cancer survivors in the United Kingdom. Eur J Cancer Care (Engl). 2022;31(6):e13722. Epub 2022/10/19. doi: 10.1111/ecc.13722 . 36255032 PMC9787781

[68] Pilon D, LaMori J, Rossi C, Durkin M, Ghelerter I, Ke X, et al. Medication adherence among patients with advanced prostate cancer using oral therapies. Future Oncology. 2022;18(2):231–43. doi: 10.2217/fon-2021-0992 . Language: English. Entry Date: 20220318. Revision Date: 20220318. Publication Type: journal article. Journal Subset: Biomedical. 34730001

[69] Tinari N, Fanizza C, Romero M, Gambale E, Moscetti L, Vaccaro A, et al. Identification of subgroups of early breast cancer patients at high risk of nonadherence to adjuvant hormone therapy: results of an Italian survey. Clin Breast Cancer. 2015;15(2):e131–7. doi: 10.1016/j.clbc.2014.10.005 25454738

[70] Di Nitto M, Sollazzo F, Biagioli V, Torino F, Alvaro R, Vellone E, et al. Self-care behaviours in older adults treated with oral anticancer agents: A qualitative descriptive study. European Journal of Oncology Nursing. 2022;58:102139. doi: 10.1016/j.ejon.2022.102139 35489295

[71] Jin J, Sklar GE, Min Sen Oh V, Chuen Li S. Factors affecting therapeutic compliance: A review from the patient’s perspective. Therapeutics and clinical risk management. 2008;4(1):269–86. doi: 10.2147/tcrm.s1458 18728716 PMC2503662

[72] Zhang NJ, Terry A, McHorney CA. Impact of health literacy on medication adherence: a systematic review and meta-analysis. Annals of Pharmacotherapy. 2014;48(6):741–51. doi: 10.1177/1060028014526562 24619949

[73] Prely H, Herledan C, Caffin AG, Baudouin A, Larbre V, Maire M, et al. Real-life drug—drug and herb—drug interactions in outpatients taking oral anticancer drugs: comparison with databases. Journal of Cancer Research and Clinical Oncology. 2022;148(3):707–18. doi: 10.1007/s00432-021-03645-z 33914124 PMC11800911

[74] Jacobs JM, Ream ME, Pensak N, Nisotel LE, Fishbein JN, MacDonald JJ, et al. Patient Experiences With Oral Chemotherapy: Adherence, Symptoms, and Quality of Life. J Natl Compr Canc Netw. 2019;17(3):221–8. Epub 2019/03/14. doi: 10.6004/jnccn.2018.7098 . 30865917 PMC6626621

[75] Higano CS, Hafron J. Adherence With Oral Anticancer Therapies: Clinical Trial vs Real-world Experiences With a Focus on Prostate Cancer. Journal of Urology. 2023;209(3):485–93. doi: 10.1097/JU.0000000000003081 36472138 PMC12721669

[76] Moon Z, Moss-Morris R, Hunter MS, Carlisle S, Hughes LD. Barriers and facilitators of adjuvant hormone therapy adherence and persistence in women with breast cancer: a systematic review. Patient Prefer Adherence. 2017;11:305–22. Epub 2017/03/07. doi: 10.2147/PPA.S126651 . 28260867 PMC5328144

[77] Mir O, Ferrua M, Fourcade A, Mathivon D, Duflot-Boukobza A, Dumont S, et al. Digital remote monitoring plus usual care versus usual care in patients treated with oral anticancer agents: the randomized phase 3 CAPRI trial. Nature Medicine. 2022;28(6):1224–31. doi: 10.1038/s41591-022-01788-1 35469070

[78] Stout NL, Wagner SS. Antineoplastic therapy side effects and polypharmacy in older adults with cancer. Top Geriatr Rehabil. 2019;35(1):15–30. Epub 2019/04/24. doi: 10.1097/TGR.0000000000000212 . 31011239 PMC6474376

[79] Moody JA, Ahmed K, Yap T, Minhas S, Shabbir M. Fertility managment in testicular cancer: the need to establish a standardized and evidence-based patient-centric pathway. BJU International. 2019;123(1):160–72. doi: 10.1111/bju.14455 29920910

[80] Komatsu H, Yagasaki K, Yamaguchi T, Mori A, Kawano H, Minamoto N, et al. Effects of a nurse-led medication self-management programme in women with oral treatments for metastatic breast cancer: A mixed-method randomised controlled trial. European Journal of Oncology Nursing. 2020;47:101780. doi: 10.1016/j.ejon.2020.101780 32674036

[81] Chin CH, Tseng LM, Chao TC, Wang TJ, Wu SF, Liang SY. Self-care as a mediator between symptom-management self-efficacy and quality of life in women with breast cancer. PLOS ONE. 2021;16(2). doi: 10.1371/journal.pone.0246430 33539460 PMC7861359

[82] Wheeler SB, Spees LP, Jackson BE, Baggett CD, Wilson LE, Greiner MA, et al. Patterns and Predictors of Oral Anticancer Agent Use in Diverse Patients With Metastatic Renal Cell Carcinoma. JCO Oncol Pract. 2021;17(12):e1895–e904. doi: 10.1200/OP.20.01082 34138665 PMC8678030

[83] Mutz J, Choudhury U, Zhao J, Dregan A. Frailty in individuals with depression, bipolar disorder and anxiety disorders: longitudinal analyses of all-cause mortality. BMC Medicine. 2022;20(1):274. doi: 10.1186/s12916-022-02474-2 36038880 PMC9425946

