# Supplementary material for: Predictors of self-care in patients with cancer treated with oral anticancer agents: A systematic review
Source: PLoS One. 2024 Sep 24;19(9):e0307838. doi: 10.1371/journal.pone.0307838 (PMC11421779; doi:10.1371/journal.pone.0307838)
Supplement: S1 Appendix — Appendix includes: Table S1A: Search Strategy. Table S1B. Characteristics of studies included. Table S1C. Methodological quality assessment of studies (cross-sectional and observational). Table S1D. Methodological quality assessment of studies (quasi-experimental). Table S1E. Assessment of methodological quality, with the jbi checklist, for cohort studies (numeric). Table S1F. Quality assessment with new version of JBI check list for RCT studies. Table S1G. Results from included studies. (DOCX) [file pone.0307838.s001.docx]

*Table S1A - Search Strategy*

| **Database Name** | **Platform** | **Date Coverage** | **Date of Search** | **# of results** |
| --- | --- | --- | --- | --- |
| 1. MEDLINE | PubMed | From 01-01-2014 | 29-6-2023 | 1,299 |
| 2. Cinahl & PsycInfo | EBSCOHost | From 01-01-2014 | 29-6-2023 | 443 |
| 3. Web of Science core collection | Clarivate | From 01-01-2014 | 29-6-2023 | 1,319 |

# 1. Pubmed

| **#** | **Search string** | **# of results** |
| --- | --- | --- |
| 1 | ("self-care"[MeSH Terms] OR "self management"[MeSH Terms] OR "self-care"[Title/Abstract] OR "self management"[Title/Abstract] OR "adherence"[Title/Abstract] OR "medication adherence"[MeSH Terms] OR "self-monitoring"[Title/Abstract]) | 258,397 |
| 2 | ((("clinical trial"[Publication Type] OR "clinical trials as topic"[MeSH Terms] OR "clinical trial"[All Fields] OR ("prospective studies"[MeSH Terms] OR prospective stud* OR "retrospective studies"[MeSH Terms] OR retrospective stud* OR "longitudinal studies"[MeSH Terms] OR longitudinal stud* OR "observational study"[Publication Type] OR "observational studies as topic"[MeSH Terms] OR "observational study"[All Fields] OR observational research OR experimental study))) OR (non randomized controlled trial)) OR ((("cross sectional studies"[MeSH Terms] OR cross sectional study) OR ("case control studies"[MeSH Terms] OR case control study)) OR ("cohort studies"[MeSH Terms] OR cohort study)) | 6,046,689 |
| 3 | ("Antineoplastic Agents"[MeSH Terms] OR "Antineoplastic Agents"[Title/Abstract] OR "anticancer medicines"[Title/Abstract] OR "anticancer medicine"[Title/Abstract] OR "chemotherapy"[Title/Abstract] OR "chemotherapies"[Title/Abstract] OR "antineoplastic drugs"[Title/Abstract] OR "antineoplastic drug"[Title/Abstract] OR "antineoplastic agents, hormonal"[MeSH Terms] OR "hormonal therapy"[Title/Abstract] OR "hormonal drugs"[Title/Abstract] OR "hormonal drug"[Title/Abstract] OR "oncolytic agent"[Title/Abstract] OR "oncolytic agents"[Title/Abstract] OR "anticancer immunotherapies"[Title/Abstract] OR "antineoplastic immunotherapy"[Title/Abstract] OR "anticancer immunotherapy"[Title/Abstract] OR "targeted drugs"[Title/Abstract] OR "targeted drug"[Title/Abstract]) | 848,030 |
| 4 | ("predictor*"[Title/Abstract] OR "predictors"[Title/Abstract] OR "risk factor*"[Title/Abstract] OR "self-care determinants"[Title/Abstract] OR "cause*"[Title/Abstract] OR "benefit*"[Title/Abstract] OR "effect*"[Title/Abstract] OR "impact"[Title/Abstract] OR "outcome"[Title/Abstract] OR "outcomes"[Title/Abstract] OR "adverse events"[Title/Abstract] OR "adverse event"[Title/Abstract] OR "Patient Outcome Assessment"[MeSH Terms] OR "Patient Outcome Assessment"[All Fields] OR "patient centered outcome research"[All Fields]) | 13,004,995 |
| 5 | 1 AND 2 AND 3 AND 4 | 1,811 |
| 6 | 1 AND 2 AND 3 AND 4 and date limit from 2014 | 1,299 |
| 7 |  |  |
| 8 |  |  |
| 9 |  |  |
| 10 |  |  |

# 2. Cinahl & PsychInfo

| **#** | **Search string** | **# of results** |
| --- | --- | --- |
| 1 | MH ("Self Care+" OR "Self-Management") OR TI ("self-care" OR selfcare OR "self-management" OR adherence OR “self-monitoring” ) OR AB ("self-care" OR selfcare OR "self-management" OR adherence OR “self-monitoring”) | 195,515 |
| 2 | ( ( rct or clinical trial* or randomized controlled trial* or controlled clinical trial* ) OR non randomized clinical trial* ) OR ( ( longitudinal stud* OR retrospective stud* OR prospective stud* OR observational stud* or observational research ) OR ( cross sectional stud* or cross-sectional stud* ) OR ( case control stud* or case-control stud* ) OR ( cohort stud* or case control stud* or experimental stud* ) ) | 1,963,414 |
| 3 | TI ( predictor* or "risk factor" or cause* or predisposition or determinant* OR "self-care determinants" or cause) OR TI ( outcome* or benefit* or effect* or impact or “adverse event” or “adverse events” OR "patient outcome assessment" OR "patient centered outcome research") OR AB ( predictor* or "risk factor" or cause* or predisposition or determinant* OR "self-care determinants") OR AB ( outcome* or benefit* or effect* or impact or “adverse event” or “adverse events” OR "patient outcome assessment" OR "patient centered outcome research") | 5,074,535 |
| 4 | TI ("Antineoplastic Agents" OR "anticancer medicines" OR "anticancer medicine" OR chemotherapy OR chemotherapies OR "antineoplastic drugs" OR "antineoplastic drug" OR "hormonal therapy" OR "hormonal drugs" OR "hormonal drug" OR "oncolytic agent" OR "oncolytic agents" OR "anticancer immunotherapies" OR "antineoplastic immunotherapy" OR "anticancer immunotherapy" OR "targeted drugs" OR "targeted drug") OR AB ("Antineoplastic Agents" OR "anticancer medicines" OR "anticancer medicine" OR chemotherapy OR chemotherapies OR "antineoplastic drugs" OR "antineoplastic drug" OR "hormonal therapy" OR "hormonal drugs" OR "hormonal drug" OR "oncolytic agent" OR "oncolytic agents" OR "anticancer immunotherapies" OR "antineoplastic immunotherapy" OR "anticancer immunotherapy" OR "targeted drugs" OR "targeted drug") | 94,105 |
| 5 | 1 AND 2 AND 3 AND 4 | 655 |
| 6 | 1 AND 2 AND 3 AND 4 and date limit from 2014 | 443 |
| 7 |  |  |
| 8 |  |  |
| 9 |  |  |
| 10 |  |  |

# 2. Web of Science

| **#** | **Search string** | **# of results** |
| --- | --- | --- |
| 1 | TS=("self-care" OR selfcare OR "self-management" OR adherence OR “self-monitoring” ) | 276,971 |
| 2 | TS=(( ( rct or clinical trial* or randomized controlled trial* or controlled clinical trial* ) OR non randomized clinical trial* ) OR ( (longitudinal stud* OR retrospective stud* OR prospective stud* OR observational stud* or observational research ) OR ( cross sectional stud* or cross-sectional stud* ) OR ( case control stud* or case-control stud* ) OR ( cohort stud* or case control stud* or experimental stud* ) )) | 5,366,063 |
| 3 | TS=(predictor* or "risk factor" or cause* or predisposition or determinant* OR "self-care determinants" or cause OR outcome* or benefit* or effect* or impact or “adverse event” or “adverse events” OR "patient outcome assessment" OR "patient centered outcome research") | 22,315,408 |
| 4 | TS=("Antineoplastic Agents" OR "anticancer medicines" OR "anticancer medicine" OR chemotherapy OR chemotherapies OR "antineoplastic drugs" OR "antineoplastic drug" OR "hormonal therapy" OR "hormonal drugs" OR "hormonal drug" OR "oncolytic agent" OR "oncolytic agents" OR "anticancer immunotherapies" OR "antineoplastic immunotherapy" OR "anticancer immunotherapy" OR "targeted drugs" OR "targeted drug") | 573,957 |
| 5 | 1 AND 2 AND 3 AND 4 | 1,695 |
| 6 | 1 AND 2 AND 3 AND 4 and date limit from 2014 | 1,319 |

*Table S1B- Characteristics of studies included*

| **Authors, Year, Country** | **Design** | **Sample population** | **Name of the anticancer** | **Main themes and findings related to self-care behaviours** | **JBI’s critical appraisal** |
| --- | --- | --- | --- | --- | --- |
| Ali et al., 2017  Singapore | Cross-sectional | Total of 157 women with breast cancer | Tamoxifen or AIs | - Presence of one or more comorbidities predict high adherence to OAA (OR=2.60, CI=1.208–5.593). | 100% |
| Arriola et al., 2014  USA | Cross-sectional | Total of 200 women with breast cancer | Tamoxifen or AIs | - Frequency of physician communication was significantly associated with OAA adherence (p < .05). | 100% |
| Bender et al., 2014  Pennsylvania | Longitudinal | Total of 91  women with breast cancer | Tamoxifen | - Higher pretherapy levels of depressive and anxiety were associated with lower adherence; - Nonadherence to OAA was associated with cognitive symptoms (p < 0.05), musculoskeletal pain (p < 0.05), weight concerns (p < 0.01), and gynecologic symptoms (p < 0.01). | 37.5% |
| Blanchette et al., 2019  Canada | Retrospective | Total of 5,692 woman with breast cancer | Tamoxifen or AIs | - Lower adherence were associated with increased age (OR=1.03, CI=1.02–1.05; - High adherence was associated with previous use of adjuvant chemotherapy (OR=0.42, CI=0.30–0.59). | 100% |
| Bourmaud et al., 2015  France | Cohort | Total of 38 patients with  colorectal cancer or breast cancer | Capecitabine | Adherence was associated with patients (p = 0.049):   - retired; - with a regular life; - surrounded by a relative’s attention to drug adherence; - with a low educational level.   Lower adherence was associated:   - highly educated; - patients with an irregular, active life. | 45% |
| Bradley et al., 2015  USA | Cohort | Total of 1536  women with breast cancer | Tamoxifen or AIs | - Women who had prescription drug coverage were more likely to initiate and continue OAA (OR=2.91, CI=1.24–6.84; OR=2.23; CI=0.99–5.05, p = 0.0543); - The lowest income women were also less likely to continue OAA relative to women with an annual household income that exceeded $70,000 (OR=0.55; CI 0.29–1.04). | 82% |
| Brett et al., 2018  UK | Cross-sectional | Total of 543  women with breast cancer | Tamoxifen or AIs | Predictors of non-adherence:   - side effects (OR=4.383, CI =.601–12.002, p < .04); - Beliefs about Medicine Concerns (OR=1.181, CI 1.033–1.350, p < .015). | 75% |
| Bright et al., 2016  USA | Cohort | Total of 1371 women with breast cancer | Tamoxifen or AIs | - Any barrier to the OAA adherence was significantly associated with nonadherence (p 0.001); - reporting the presence of a side effect was associated to nonadherence (p 0.05); - cognitive facilitators were associated with adherence (p 0.05). | 64% |
| Cakmak & Uncu, 2020  India | Cross‐sectional | A total of 100 patients with  Breast cancer, Colon cancer, Gastric cancer, Brain cancer, Rectum cancer | Capecitabine, Tamoksifen, Sunitinibe, Temozolomide,Letrazol,Regorafenib | - There was a positive and strong correlation between OAA adherence and health literacy (p = 0.000). | 87.5% |
| Chin et al., 2019  USA | Cohort | Total of 6900  women with breast cancer. | Tamoxifen or AIs | - Higher median monthly copayment was significantly associated with a higher risk of non-adherence (p<0.001); - median total out-of-pocket was significantly associated with a higher risk of non-adherence (p<0.001). | 73% |
| Corter et al., 2018  USA | Cohort | Total of 125  women with breast cancer | Tamoxifen or AI | - Lower illness coherence scores and the presence of comorbidity were predictors of nonadherence. | 73% |
| Dinam et al., 2022 | Retrospective | Total of 905 patients with Metastatic Renal Cell  Carcinoma (mRCC) | Sorafenib, Sunitinib, Pazopanib, Everolimus, and Axitinib | Factors associated with lower adherence:   - living within an impoverished neighbourhood (CI=0.33-0.74); - taking drugs with out-of-pocket costs >$200 (CI=0.47-0.98); - living in the northeast compared with the west (CI=0.42-0.95); - aged 76-80 (CI=0.37-0.78) or aged 81+ (CI=0.35-0.85); - taking pazopanib (CI=0.45-0.90) and sorafenib (CI=0.31-0.90) compared with patients taking sunitinib. - Males had higher prevalence of adherence than females (CI=1.03-1.88). | 75% |
| Font et al., 2017  UK | Observational | Total of 119 patients with  rectal cancer | Capecitabine | - There was a significant association between adherence and depression and anxiety together. | 87.5% |
| Haskins et al.,2019  German | Cohort | Total of 21894  women with breast cancer | Tamoxifen or AIs | - Patients aged 85–94 at diagnosis had 1.2% greater adherence compered to younger patients; - Asian patients had 1.2% greater adherence than White patients, and “other” races saw a 3.9% reduction; - adherence decreased with advancing stage (stage III −1.2%; stage IV −4.9%); - Patients more recently diagnosed with cancer had higher adherence rates (+8.4% 2013 vs 2007); - With each consecutive year of use, adherence improved (year 2 +6.6%; year 3 +8.8%; year 4 +11.8%; year 5 +13.0%). | 91% |
| Haskins et al., 2020  Netherlands | Cohort | Total of 21894  women with breast cancer | SERS or AIs | - Out-of-pocket costs increased adherence decreased (CI = −1.8, −1.1); - patients without bipolar and psychotic disorder diagnoses, prior medication use, and pharmacy visits are positively associated with adherence. | 91% |
| Hefner et al., 2018  UK | Cross-sectional | Total of 64 patients with  Colorectal cancer, Stomach cancer, Breast cancer, Pancreatic cancer | Capecitabine | - Beliefs about medicine (CI=1.090–1.475) and satisfaction with information about medicine (CI=1.010–1.551) were predictors of adherence. | 87.5% |
| Hess et al., 2017  USA | Longitudinal | Total of 1452 patients with  Non-Small Cell Lung Cancer | Erlotinib | Predictors of adherence:   - Receipt of low-income subsidy; - previous use of intravenous chemotherapy; - health care costs and patient out-of-pocket. | 100% |
| Hirao et al., 2017  Japan | Cross-sectional | Total of 117 patients with  gastroenterological cancer | Capecitabine, Sorafenib, Imatinib | Predictors of non-adherence:   - having diarrhea (CI = 1.13–9.34); - experiencing pain (CI = 0.05–0.55); - taking oral chemotherapy medication every 8h (CI = 1.71–17.81); - diminished sense of priority for medication (CI = 1.21–1.63). | 100% |
| Hu et al., 2022  USA | Retrospective | Total of 559 women with breast cancer | Tamoxifen or AIs | - being Black was associated with 6.5 percentage points higher adherence than being White. | 100% |
| Hwang et al., 2020  Netherlands | Retrospective | Total of 338  women with breast cancer | Tamoxifen or AIs | Associated with adherence:   - • Compared to white/non-Hispanics, patients who were Asian/non-Hispanic or white/Hispanic were less likely to be adherent (Asian/non-Hispanic: CI=0.11-0.82; white/Hispanic: CI=0.11-0.64); - Patients prescribed AIs were more likely to be adherent compared to patients prescribed tamoxifen (CI=1.02-4.14); - Patients prescribed OAA for 3 to 5 years had lower adherence compared to patients given OAA for 2 years or less (CI=0.09-0.91). | 100% |
| Iacorossi et al., 2016  Italy | Cross-sectional | Total of 151 women with breast cancer | Tamoxifen or AIs | Significant correlations were found between adherence and:   - marital status (married/living in defacto relationships p=0.04); - side effect of difficulty in concentrating and memory (p=0.01); - education (p=0.05).   Non-significant differences of adherence were found at:   - type of oral OAA (Tamoxifen vs AIs; p=0.52); - between age groups (p=0.38). | 75% |
| Karavites et al., 2017  USA | Cohort | A total of 368 women with  ductal carcinoma in situ | Tamoxifen | - Complete local therapy (breast conservation surgery (BCS) or breast radiotherapy (RT)) was a significant predictor; - Insured patients were more likely to adhere to tamoxifen: (OR=6.03, CI=2.60-13.98, p<0.01), and (OR=5.64, CI=2.25-14.14, p<0.01) in the subset of patients who had breast-conserving surgery. . | 82% |
| Keim-Malpass et al., 2020  USA | Cohort | A total of 12210 woman with breast cancer | Tamoxifen or AIs | - The woman who had radiotherapy omitted were statistically less likely to adhere to OAA; - 85 years of age or older were 55% less likely to be adherent to OAA than the women 70–75 years of age (CI= 0.39–0.53). | 82% |
| Koni et al., 2023  Palestine | Cross-sectional | Total of 106 women with breast cancer | Tamoxifen or AIs | - the side effects (p = 0.013) and global satisfaction (p = 0.018) were predictors of OAA. | 87.5% |
| Ma et al., 2020  USA | Cohort | Total of 10,905  women with breast cancer. | Tamoxifen or AIs | - Low Income Subsidy (LIS) beneficiaries were 11.4% higher than among non-LIS beneficiaries (p<0.001). Non-LIS beneficiaries had an overall decreasing trend of adherence (p < 0.001) | 82% |
| McGuinness et al., 2022  UK | Observational | Total of 2,001 women with breast cancer | Tamoxifen or AIs | - Women from minority ethnic groups had a significantly higher risk of non-adherence than women who were White British (OR= 1.50, p = 0.03). | 100% |
| Murphy et al., 2021  USA | RTC | Total of 1,943 patients with renal cell carcinoma. | Sunitinib or Sorafenib | - Non-adherence was statistically significantly associated with race/ethnicity; - Type of treatment; - Skin rash. | 69% |
| Neugut et al., 2016  USA | Cohort | Total of 21,255 woman with breast cancer. | Tamoxifen or AIs | - The woman who used 1 or more medication prior to OAA and were adherent had a higher probability to adherence to OAA. | 82% |
| Neuner et al., 2015  USA | Quasi-experimental | Total of 16,462 women with breast cancer. | AIs | - Adherence was 5.4% higher after generic anastrozole was introduced and 11% higher after generic letrozole/exemestane was introduced. - Subsidy recipients had higher adherence | 62.5% |
| Oke et al., 2022  Texas | Cohort | Total of 451 male with breast cancer | Tamoxifen | Factors associated with high adherence:   - Age of 76 to 80 years (OR=2.75, CI=1.26- 6.02) - residing in metro (OR=2.40, CI=1.20- 4.82) or less urban areas (OR=8.54, CI=1.81- 40.3); - Charlson comorbidity score of ≥2 (OR=0.46, CI= 0.22- 0.97) was a significant predictor of lower adherence. | 82% |
| Pan et al., 2018  German | Prospective | Total of 116 women with breast cancer. | Tamoxifen or AIs | Predictors of adherence:   - Side-effect (CI=0.08- 0.81, p = 0.02); - necessity–concern beliefs (CI=1.11-3.72, p = 0.02). | 75% |
| Pilon et al., 2022  USA | Observational | A total of 27,262 patients with prostate cancer | Apalutamide, Enzalutamide or Abiraterone acetate | Factors associated with poor adherence:   - Aged ≥75 years; - black race; - chemotherapy use; - higher pharmacy paid. | 100% |
| Pourcelot et al., 2018  France | Cross-sectional |  | Tamoxifen or AIs | Risk factors for non-adherence:   - 2 medications to treat comorbidities (p= 0.003); - age less than 65 years (p= 0.008); - patient management in a university hospital setting (p= 0.014). | 100% |
| Rassy et al., 2022  France | Cohort | Total of 10,863 women with breast cancer. | Tamoxifen or AIs | - No statistically significant associations were found between potential-drug-drug-interaction and adherence. | 73% |
| Stahlschmidt et al., 2019  German | Cross-sectional | Total of 58 women with breast cancer. | Tamoxifen or AIs | - Women with low/medium adherence had more systemic side effects than women with high adherence (p = 0.0346). - Half of the women in stage I of the disease had high adherence and 42% of women with stage III/IV had low/medium adherence (p = 0.0229). - There was no significant difference in adherence levels between tamoxifen and AIs (p = 0.4759). | 37.5% |
| Sugita et al., 2016  Swiss | Retrospective | A total of 50 patients with  Colorectal Cancer | Trifluridine and Tipiracil Hydrochloride | - ECOG performance status ≥1 (CI = 2.75– 236.19; p = 0.01); - number of prior regimens ≥4 (CI = 1.24–106.43; p = 0.03). | 45% |
| Tinari et al., 2015  Italy | Observational |  | Tamoxifen or AIs | - Patients 45 years of age (OR=2.56, CI=1.03-6.34) and those between 61 and 70 years of age (OR=1.93, CI=1.09-3.4) had the greatest odds of being nonadherent; - Retired patients showed the lowest percentage of nonadherers and the greatest odds of nonadherence was observed for housewives (OR=1.92, CI=1.17-3.15); - the risk of nonadherence was greater among patients who switched from tamoxifen to aromatase inhibitors. | 55% |
| Vacher et al., 2020  German | Prospective | A total of 65 patients with  colorectal or breast cancer | Capecitabine or Lapatinib | Factors influencing the adherence:   - hand-foot syndrome (p = 0.023); - toxicity grade (p = 0.024); - the type of treatment received (p = 0.046); - diarrhea (p = 0.022) - the number of toxicities (p = 0.028) | 100% |
| Valachis et al., 2016  German | Cohort | Total of 18 432 women with breast cancer | Tamoxifen or AIs | - SSRI use was associated with a higher risk for low adherence to oral endocrine therapy. | 82% |
| Winn et al., 2019  USA | Cohort | Total of 3344 women with breast cancer | Anastrozole, letrozole or exemestane | - Comparing patients that did and did not receive a subsidy the author founded no overall effect of generic introduction. | 100% |
| Yan et al., 2023  China | Retrospective | Total of 706 women with breast cancer | Tamoxifen or AIs | - Lumpectomy (OR=1.75, CI=1.07–2.83, p= 0.024) and Tamoxifen or AIs (OR=2.25, CI01.14–4.24, p = 0.015) were the two influencing factors that decreased adherence. | 100% |
| Yang et al., 2021  China | RTC | Total of 100 patients with prostate cancer | Hormono-Therapy | - The healthy behaviour adherence was also significantly better than that of the control group (p< 0.05) except for the medication adherence and follow up adherence dimensions (p > 0.05) | 54% |
| Yuan et al., 2020  German | Cohort | Total of 552  women with breast cancer | Tamoxifen or AIs | Associate with higher adherence to OAAs:   - PR positive (CI=1.09–2.85; p = 0.025); - tumor size larger than 50 mm (CI=1.05–3.75; p = 0.035); - patients who lived in the western USA were less likely to adhere to OAAs than those living in the northeast (CI=0.36–0.94; p = 0.028); - patients with comorbidity score 1 or more were more adherent to OAAs than patients with no comorbidity (comorbidity score 1: OR=1.69, CI=1.02–2.80; p = 0.043); - the type of OAAs (tamoxifen vs. AIs) made no difference in adherence. | 91% |
| Zahrina et al., 2014  Malaysia | Cross-sectional | A total of 130 patients with Colorectal and Breast Cancer | Capecitabine | Factors of adherence:   - being female (CI=0.61-2.99; p=0.003); - satisfaction score (CI=0.06-1.46; p value=0.035); - presence of nausea or vomiting (CI=1.12-3.48; p <0.001) and other side effects (CI=0.24-2.65; p=0.019). | 100% |
| Zeidan et al., 2016  Netherlands | Cohort | Total of 327 women with breast cancer | Tamoxifen | - A significant number of patients considered discontinuing tamoxifen because of the side effects (p=0.001), but this did not translate into discontinuation or non-adherence. | 55% |

***Table S1C - Methodological quality assessment of studies (cross-sectional and observational)***

| **Author & Year** | **Were the criteria for inclusion in the sample clearly defined?** | **Were the study subjects and the setting described in detail?** | **Was the exposure measured in a valid and reliable way?** | **Were objective, standard criteria used for measurement of the condition?** | **Were confounding factors identified?** | **Were strategies to deal with confounding factors stated?** | **Were the outcomes measured in a valid and reliable way?** | **Was appropriate statistical analysis used?** | **%** |
| --- | --- | --- | --- | --- | --- | --- | --- | --- | --- |
| Ali et al., 2017 | 1 | 1 | 1 | 1 | 1 | 1 | 1 | 1 | 100% |
| Arriola et al., 2014 | 1 | 1 | 1 | 1 | 1 | 1 | 1 | 1 | 100% |
| Bender et al., 2014 | 1 | 1 | 0 | 1 | 0 | 0 | 0 | 0 | 37.5% |
| Blanchette et al., 2015 | 1 | 1 | 1 | 1 | 1 | 1 | 1 | 1 | 100% |
| Brett et al., 2018 | 0 | 0 | 1 | 1 | 1 | 1 | 1 | 1 | 75% |
| Cakmak & Uncu, 2020 | 0 | 1 | 1 | 1 | 1 | 1 | 1 | 1 | 87.5% |
| Dinam et al., 2022 | 1 | 0 | 1 | 1 | 1 | 1 | 1 | 0 | 75% |
| Font et al., 2017 | 1 | 1 | 0 | 1 | 1 | 1 | 1 | 1 | 87.5% |
| Hefner et al., 2018 | 0 | 1 | 1 | 1 | 1 | 1 | 1 | 1 | 87.5% |
| Hess et al., 2017 | 1 | 1 | 1 | 1 | 1 | 1 | 1 | 1 | 100% |
| Hirao et al., 2017 | 1 | 1 | 1 | 1 | 1 | 1 | 1 | 1 | 100% |
| Hu et al., 2022 | 1 | 1 | 1 | 1 | 1 | 1 | 1 | 1 | 100% |
| Hwang et al., 2020 | 1 | 1 | 1 | 1 | 1 | 1 | 1 | 1 | 100% |
| Koni et al., 2023 | 1 | 1 | 1 | 1 | 1 | 1 | 0 | 1 | 87.5% |
| Iacorossi et al., 2016 | 1 | 1 | 1 | 1 | 0 | 0 | 1 | 1 | 75% |
| McGuinness et al., 2022 | 1 | 1 | 1 | 1 | 1 | 1 | 1 | 1 | 100% |
| Pan et al., 2018 | 0 | 0 | 1 | 1 | 1 | 1 | 1 | 1 | 75% |
| Pilon et al., 2022 | 1 | 1 | 1 | 1 | 1 | 1 | 1 | 1 | 100% |
| Pourcelot et al., 2018 | 1 | 1 | 1 | 1 | 1 | 1 | 1 | 1 | 100% |
| Stahlschmidt et al., 2019 | 1 | 1 | 0 | 1 | 0 | 0 | 0 | 0 | 37.5% |
| Tinari et al., 2015 | 0 | 1 | 1 | 1 | 1 | 1 | 1 | 1 | 87.5% |
| Vacher et al., 2020 | 1 | 1 | 1 | 1 | 1 | 1 | 1 | 1 | 100% |
| Yan et al., 2023 | 1 | 1 | 1 | 1 | 1 | 1 | 1 | 1 | 100% |
| Zahrina et al., 2014 | 1 | 1 | 1 | 1 | 1 | 1 | 1 | 1 | 100% |
| **Quality assessment** |  |  |  |  |  |  |  |  |  |
|  | | |  |  |  |  |  |  |  |

***Table S1D - Methodological quality assessment of studies (quasi-experimental)***

| **Author & Year** | **Is it clear in the study what is the ‘cause’ and what is the ‘effect’** | **Were the participants included in any comparisons similar?** | **Were the participants included in any comparisons receiving similar treatment/care, other than the exposure or intervention of interest?** | **Was there a control group?** | **Were there multiple measurements of the outcome both pre and post the intervention/exposure?** | **Was follow up complete and if not, were differences between groups in terms of their follow up adequately described and analyzed?** | **Were the outcomes of participants included in any comparisons measured in the same way?** | **Were outcomes measured in a reliable way?** | **Was appropriate statistical analysis used?** | **%** |
| --- | --- | --- | --- | --- | --- | --- | --- | --- | --- | --- |
| Neuner et al., 2015 | 0 | 1 | 1 | 0 | 0 | 0 | 1 | 1 | 1 | 62.5% |
| **Quality assessment** |  |  |  |  |  |  |  |  |  |  |

| Tab. S1E Assessment of methodological quality, with the jbi check-list, for cohort studies (numeric) | | | | | | | | | | | | | |
| --- | --- | --- | --- | --- | --- | --- | --- | --- | --- | --- | --- | --- | --- |
| Author, year | Study design | item 1  Were the two groups similar and recruited from the same population? | item 2  Were the exposures measured similarly to assign people to both exposed and unexposed groups? | item 3  Was the exposure measured in a valid and reliable way? | item 4  Were confounding factors identified? | item 5  Were strategies to deal with confounding factors stated? | item 6  Were the groups/participants free of the outcome at the start of the study (or at the moment of exposure)? | item 7  Were the outcomes measured in a valid and reliable way? | item 8  Was the follow up time reported and sufficient to be long enough for outcomes to occur? | item 9  Was follow up complete, and if not, were the reasons to loss to follow up described and explored? | item 10  Were strategies to address incomplete follow up utilized? | item 11  Was appropriate statistical analysis used? | Total score  (%) |
| Bourmaud, 2015 | COHORT | 1 | 0 | 1 | 0 | 0 | 1 | 0 | 1 | 0 | 0 | 1 | 45% |
| Bradley, 2015 | COHORT | 1 | 1 | 1 | 1 | 1 | 1 | 1 | 1 | 0 | 0 | 1 | 82% |
| Bright, 2016 | COHORT | 1 | 1 | 1 | 0 | 0 | 1 | 1 | 1 | 0 | 0 | 1 | 64% |
| Chinn, 2019 | COHORT | 1 | 1 | 1 | 0 | 1 | 1 | 1 | 1 | 0 | 0 | 1 | 73% |
| Corter, 2018 | COHORT | 1 | 1 | 1 | 0 | 0 | 1 | 0 | 1 | 0 | 0 | 1 | 73% |
| Haskins, 2019 | COHORT | 1 | 1 | 1 | 1 | 1 | 1 | 1 | 1 | 1 | 0 | 1 | 91% |
| Haskins, 2020 | COHORT | 1 | 1 | 1 | 1 | 1 | 1 | 1 | 1 | 1 | 0 | 1 | 91% |
| Karavites, 2017 | COHORT | 1 | 1 | 1 | 1 | 1 | 1 | 1 | 1 | 0 | 0 | 1 | 82% |
| Keim-Malpass et al., 2020 | COHORT | 1 | 1 | 1 | 1 | 1 | 1 | 1 | 1 | 0 | 0 | 1 | 82% |
| Ma, 2020 | COHORT | 1 | 1 | 1 | 1 | 1 | 1 | 1 | 1 | 0 | 0 | 1 | 82% |
| Neugut, 2016 | COHORT | 1 | 1 | 1 | 0 | 1 | 1 | 1 | 1 | 0 | 1 | 1 | 82% |
| Oke,2022 | COHORT | 1 | 1 | 1 | 1 | 1 | 1 | 1 | 1 | 0 | 0 | 1 | 82% |
| Rassy, 2022 | COHORT | 1 | 1 | 1 | 1 | 1 | 0 | 1 | 1 | N/A | N/A | 1 | 73% |
| Sugita, 2016 | COHORT | 1 | 0 | 1 | 1 | 1 | 0 | 0 | 0 | 0 | 0 | 1 | 45% |
| Valachis, 2016 | COHORT | 1 | 1 | 1 | 1 | 1 | 0 | 0 | 1 | 1 | 1 | 1 | 82% |
| Winn, 2019 | COHORT | 1 | 1 | 1 | 1 | 1 | 1 | 1 | 1 | 1 | 1 | 1 | 100% |
| Yuan, 2020 | COHORT | 1 | 0 | 1 | 1 | 1 | 1 | 1 | 1 | 1 | 1 | 1 | 91% |
| Zeidan, 2016 | COHORT | 1 | 1 | 0 | 1 | 0 | 1 | 0 | 1 | 0 | 0 | 1 | 55% |

**Table S1F: Quality assessment with new version of JBI check list for RCT studies.**

| **Study Author: Murphy et al.** | | | **Study Title: Adherence to oral therapies among patients with renal cell carcinoma: Post hoc analysis of the ECOG-ACRIN E2805 trial** | | | | | | | | | | | **Study Year: 2021** | | | | | |
| --- | --- | --- | --- | --- | --- | --- | --- | --- | --- | --- | --- | --- | --- | --- | --- | --- | --- | --- | --- |
|  | | | | | | | | | | | | | | | | | | | |
| **Internal Validity** | | | | | | | | **Choice - Comments/Justification** | **Yes** | **No** | | | | | **Unclear** | **N/A** | | | |
| **Bias related to selection and allocation** | | | | | | | | | | | | | | | | | | | |
| **+** | **Was true randomization used for assignment of participants to treatment groups?** | | | | | | |  |  | |  | | | |  |  | | | |
| **2** | **Was allocation to treatment groups concealed?** | | | | | | |  |  | |  | | | |  |  | | | |
| **3** | **Were treatment groups similar at the baseline?** | | | | | | |  |  | |  | | | |  |  | | | |
| **Bias related to administration of intervention/exposure** | | | | | | | | | | | | | | | | | | | |
| **4** | **Were participants blind to treatment assignment?** | | | | | | |  |  | |  | | | |  |  | | | |
| **5** | **Were those delivering the treatment blind to treatment assignment?** | | | | | | |  |  | |  | | | |  |  | | | |
| **6** | **Were treatment groups treated identically other than the intervention of interest?** | | | | | | |  |  | |  | | | |  |  | | | |
| **Bias related to assessment, detection and measurement of the outcome** | | | | | | | | | | | | | | | | | | | |
| **7** | **Were outcome assessors blind to treatment assignment?** | | | | | | |  | **Yes** | | **No** | | | | **Unclear** | **N/A** | | | |
|  | **Outcome 1** | | | | | | |  |  | |  | | | |  |  | | | |
|  | **Outcome 2-7** | | | | | | |  |  | |  | | | |  |  | | | |
|  |  | | | | | | |  |  | |  | | | |  |  | | | |
| **8** | **Were outcomes measured in the same way for treatment groups?** | | | | | | |  | **Yes** | | **No** | | | | **Unclear** | **N/A** | | | |
|  | **Outcome +1** | | | | | | |  |  | |  | | | |  |  | | | |
|  | **Outcome 2-7** | | | | | | |  |  | |  | | | |  |  | | | |
|  |  | | | | | | |  |  | |  | | | |  |  | | | |
| **9** | **Were outcomes measured in a reliable way** | | | | | | |  | **Yes** | | **No** | | | | **Unclear** | **N/A** | | | |
|  | **Outcome 1** | | | | | | |  |  | |  | | | |  |  | | | |
|  | **Outcome 2-7** | | | | | | |  |  | |  | | | |  |  | | | |
|  |  | | | | | | |  |  | | | | | | | | | | |
| **Bias related to participant retention** | | | | | | | | | | | | | | | | | | | |
| **+0** | **Was follow-up complete and if not, were differences between groups in terms of their follow-up adequately described and analyzed?** | | | | | | |  |  | | | | | | | | | | |
|  | **Outcome 1** | | | | | | |  | **Yes** | | **No** | | | | **Unclear** | **N/A** | | | |
|  |  | Result 1 | | | | | |  |  | |  | | | |  |  | | | |
|  |  | Result 2 | | | | | |  |  | |  | | | |  |  | | | |
|  |  | Result 3 | | | | | |  |  | |  | | | |  |  | | | |
|  |  | Result 4 | | | | | |  |  | |  | | | |  |  | | | |
|  | **Outcome 2-7** | | | | | | |  | **Yes** | | **No** | | | | **Unclear** | **N/A** | | | |
|  |  | Result 1 | | | | | |  |  | |  | | | |  |  | | | |
|  |  |  | | | | | | |  | |  | | | |  |  | | | |
|  | **Statistical Conclusion Validity** | | | | | | | |  | |  | | | |  |  | | | |
| **++** | **Were participants analyzed in the groups to which they were randomized?** | | | | | | |  |  | | | | | | | | | | |
|  | **Outcome 1** | | | |  | | |  | **Yes** | | **No** | | | | **Unclear** | **N/A** | | | |
|  |  | Result 1 | | | | | |  |  | |  | | | |  |  | | | |
|  |  | Result 2 | | | | | |  |  | |  | | | |  |  | | | |
|  |  | Result 3 | | | | | |  |  | |  | | | |  |  | | | |
|  |  | Result 4 | | | | | |  |  | |  | | | |  |  | | | |
|  | **Outcome 2-7** | | | |  | | |  | **Yes** | | **No** | | | | **Unclear** | **N/A** | | | |
|  |  | Result 1 | | | | | |  |  | |  | | | |  |  | | | |
|  |  | | | | | | | |  | | |  | | |  | |  | | |
| **+2** | **Was appropriate statistical analysis used?** | | | | | | |  |  | | |  | | |  | |  | | |
|  | **Outcome 1** | | | | | | |  | **Yes** | | | **No** | | | **Unclear** | | **N/A** | | |
|  |  | Result 1 | | | | | |  |  | | |  | | |  | |  | | |
|  |  | Result 2 | | | | | |  |  | | |  | | |  | | |  | |
|  |  | Result 3 | | | | | |  |  | | |  | | |  | | |  | |
|  | **Outcome 2-7** | | | | | | |  | **Yes** | | | **No** | | | **Unclear** | | | **N/A** | |
|  |  | Result 1 | | | | | |  |  | | |  | | |  | | |  | |
|  |  | | | | | | |  | **Yes** | | | | **No** | | **Unclear** | | | | **N/A** |
| **+3** | **Was the trial design appropriate and any deviations from the standard RCT design (individual randomization, parallel groups) accounted for in the conduct and analysis of the trial?** | | | | | | |  |  | | | |  | |  | | | |  |
| **Overall appraisal:** | | | | **Include:** | | **Exclude:** | **Seek Further Info:** | | | | | | | | | | | | |
| **Comments:** | | | | | | | | | | | | | | | | | | | |

| **Study Author: Yang et al.** | | | | | | **Study Title: The Effect of an Information Support Program on Self‑Efficacy of Prostate Cancer Patients during Hormonal Therapy** | | | | | | | **Study Year: 2021** | | | | | | | | |
| --- | --- | --- | --- | --- | --- | --- | --- | --- | --- | --- | --- | --- | --- | --- | --- | --- | --- | --- | --- | --- | --- |
|  | | | | | | | | | | | | | | | | | | | | | |
| **Internal Validity** | | | | | | | | **Choice - Comments/Justification** | **Yes** | **No** | | | | **Unclear** | | | | **N/A** | | | |
| **Bias related to selection and allocation** | | | | | | | | | | | | | | | | | | | | | |
| **+** | **Was true randomization used for assignment of participants to treatment groups?** | | | | | | |  |  |  | | | |  | | | |  | | | |
| **2** | **Was allocation to treatment groups concealed?** | | | | | | |  |  |  | | | |  | | | |  | | | |
| **3** | **Were treatment groups similar at the baseline?** | | | | | | |  |  |  | | | |  | | | |  | | | |
| **Bias related to administration of intervention/exposure** | | | | | | | | | | | | | | | | | | | | | |
| **4** | **Were participants blind to treatment assignment?** | | | | | | |  |  |  | | | |  | | | |  | | | |
| **5** | **Were those delivering the treatment blind to treatment assignment?** | | | | | | |  |  |  | | | |  | | | |  | | | |
| **6** | **Were treatment groups treated identically other than the intervention of interest?** | | | | | | |  |  |  | | | |  | | | |  | | | |
| **Bias related to assessment, detection and measurement of the outcome** | | | | | | | | | | | | | | | | | | | | | |
| **7** | **Were outcome assessors blind to treatment assignment?** | | | | | | |  | **Yes** | **No** | | | | **Unclear** | | | | **N/A** | | | |
|  | **Outcome 1** | | | | | | |  |  |  | | | |  | | | |  | | | |
|  | **Outcome 2-7** | | | | | | |  |  |  | | | |  | | | |  | | | |
|  |  | | | | | | |  |  |  | | | |  | | | |  | | | |
| **8** | **Were outcomes measured in the same way for treatment groups?** | | | | | | |  | **Yes** | **No** | | | | **Unclear** | | | | **N/A** | | | |
|  | **Outcome 1** | | | | | | |  |  |  | | | |  | | | |  | | | |
|  | **Outcome 2-7** | | | | | | |  |  |  | | | |  | | | |  | | | |
|  |  | | | | | | |  |  |  | | | |  | | | |  | | | |
| **9** | **Were outcomes measured in a reliable way** | | | | | | |  | **Yes** | **No** | | | | **Unclear** | | | | **N/A** | | | |
|  | **Outcome 1** | | | | | | |  |  |  | | | |  | | | |  | | | |
|  | **Outcome 2-7** | | | | | | |  |  |  | | | |  | | | |  | | | |
|  |  | | | | | | |  |  | | | | | | | | | | | | |
| **Bias related to participant retention** | | | | | | | | | | | | | | | | | | | | | |
| **10** | **Was follow-up complete and if not, were differences between groups in terms of their follow-up adequately described and analyzed?** | | | | | | |  |  | | | | | | | | | | | | |
|  | **Outcome 1** | | | | | | |  | **Yes** | **No** | | | | | **Unclear** | | | **N/A** | | | |
|  |  | Result 1 | | | | | |  |  |  | | | |  | | | |  | | | |
|  |  | Result 2 | | | | | |  |  |  | | | |  | | | |  | | | |
|  |  | Result 3 | | | | | |  |  |  | | | |  | | | |  | | | |
|  | **Outcome 2-7** | | | | | | |  | **Yes** | **No** | | | | **Unclear** | | | | **N/A** | | | |
|  |  | Result 1 | | | | | |  |  |  | | | |  | | | |  | | | |
|  |  |  | | | | | | |  |  | | | |  | | | |  | | | |
|  | **Statistical Conclusion Validity** | | | | | | | |  |  | | | |  | | | |  | | | |
| **11** | **Were participants analyzed in the groups to which they were randomized?** | | | | | | |  |  | | | | | | | | | | | | |
|  | **Outcome 1** | | |  | | | |  | **Yes** | **No** | | | | **Unclear** | | | | **N/A** | | | |
|  |  | Result 1 | | | | | |  |  |  | | | |  | | | |  | | | |
|  |  | Result 2 | | | | | |  |  |  | | | |  | | | |  | | | |
|  |  | Result 3 | | | | | |  |  |  | | | |  | | | |  | | | |
|  | **Outcome 2-7** | | |  | | | |  | **Yes** | **No** | | | | **Unclear** | | | | **N/A** | | | |
|  |  | Result 1 | | | | | |  |  |  | | | |  | | | |  | | | |
|  |  | | | | | | | |  | |  | | | | | |  | |  | | |
| **12** | **Was appropriate statistical analysis used?** | | | | | | |  |  | |  | | | | | |  | |  | | |
|  | **Outcome 1** | | | | | | |  | **Yes** | | **No** | | | | | | **Unclear** | | **N/A** | | |
|  |  | Result 1 (HEALTHY BEHAVIOURAL ADHERENCE) | | | | | |  |  | |  | | | | | |  | |  | | |
|  |  | Result 2 | | | | | |  |  | |  | | | | |  | | | |  | |
|  |  | Result 3 | | | | | |  |  | |  | | | | |  | | | |  | |
|  | **Outcome 2-7** | | | | | | |  | **Yes** | | **No** | | | | | **Unclear** | | | | **N/A** | |
|  |  | Result 1 | | | | | |  |  | |  | | | | |  | | | |  | |
|  |  | | | | | | |  |  | | |  | | | |  | | | | |  |
|  |  | | | | | | |  | **Yes** | | | **No** | | | | **Unclear** | | | | | **N/A** |
| **13** | **Was the trial design appropriate and any deviations from the standard RCT design (individual randomization, parallel groups) accounted for in the conduct and analysis of the trial?** | | | | | | |  |  | | |  | | | |  | | | | |  |
| **Overall appraisal:** | | | **Include:** | | **Exclude:** | | **Seek Further Info:** | | | | | | | | | | | | | | |
| **Comments:** | | | | | | | | | | | | | | | | | | | | | |

|  |  |  |  |  |  |  |  |  |
| --- | --- | --- | --- | --- | --- | --- | --- | --- |

*Table S1G – Results from included studies*

| **Theme** | **Categories** | **Predictors** | **Number of studies finding positive/negative adherence** |
| --- | --- | --- | --- |
| **Person** | **Socio-demographic Factors** | *Age* | N (1-18)  Increased age - (19-23)  Increased age + (24-29) |
|  |  | *Sex* | N(7-9, 16, 17, 29)  F + (30)  M + (20) |
|  |  | *Marital status* | N (3, 8, 14, 20, 27, 28)  + (11) |
|  |  | *Ethnicity* | N (3, 9, 12, 13, 20, 27, 29, 30)  +(10, 22, 24-26, 31-33) |
|  |  | *Education* | N (2-4, 14, 15, 18, 27, 28, 34)  Low education + (11, 35) |
|  |  | *Job* | N (1, 4)  Retired + (28, 35)  Housewives - (28) |
|  | **Knowledge** | *Health Literacy* | + (6, 36-38) |
|  | **Experience** | *Previous use of intravenous chemotherapy* | + (19)  -(9, 22) |
|  |  | *Years of endocrine therapy use* | + (24, 25) |
|  |  | *Satisfaction with healthcare providers* | N (8)  + (30, 39) |
|  |  | *Satisfaction with information about medicine* | +(8, 31, 39) |
|  |  | *Beliefs about medicine* | + (4, 8, 14, 34, 36) |
|  | **Habits** | *Drug use* | - (24, 25) |
| **Problem** | ***Comorbidities*** | *Comorbidities* | *N* (5, 9, 13, 14, 18, 20)  *+ The presence of comorbidities* (1, 19, 27, 33).  *- The absence of comorbidities (6)* |
|  | ***Psychological disorders*** | *Depression and Anxiety* | *-* (2, 7, 25) |
|  |  | *Other mental disorders* | *-* (2, 25) |
|  |  | *Anxiety* | *-* (2, 7, 14, 24, 25). |
|  | ***Condition related factors*** | *Recently diagnosed cancer* | *N*  (8, 18)  + (24) |
|  |  | *Metastatic cancer* | *N* (16, 18, 22) |
|  |  | *Performance status* | *N (17)*  *-* (2, 16) |
|  |  | *Breast-conserving surgery*  *“Complete local therapy” (RT or mastectomy)* | *N* (18, 27)  + (12) |
|  |  | *Having four or more positive lymph nodes* | *N* (8, 18) |
|  |  | *Progesterone-positive* | *N (18)*  *+ (33)* |
|  |  | *Stage* | *N* (7, 8, 10, 12, 14, 18, 27)  *-* (3, 15, 25) |
|  |  | *Tumour size* | *N* (7, 8, 12, 18)  *+ (33)* |
|  | **Other therapies** | *Use of selective serotonin reuptake inhibitors (SSRI)* | *+an overlapping period > 50% (18)* |
|  |  | *Polypharmacotherapy* | *N (1, 7, 14, 15, 40)*  *+ (25)*  *- (29)* |
|  |  | *Prior medication use* | *N (16)*  *- (41)* |
|  |  | *Prior regimens >4* | *- (42)* |
|  |  | *Radiotherapy* | *+ (12)* |
|  | **Therapy-related factors** | *Side Effects* | N (8, 16)  - (2, 4, 11, 14, 15, 17, 30, 34, 36, 39, 43, 44)  Avoidance of side effects - (1) |
|  |  | *Type of anticancer therapy* | N (1, 5, 8, 11, 13-15)  + (10, 17, 19, 20, 43)  + Aromatase Inhibitors vs Tamoxifen (10)  - Pazopanib vs Sunitinib  - Sorafenib vs Sunitinib (20)  + Capecitabine+ Lapatinib vs Capecitabine (17) |
|  |  | *Taking oral chemotherapy medication every 8 h* | - (34) |
|  |  | *Switching to another therapy* | N (10, 14)  - (28) |
|  |  | *Years of diagnosis* | N (8, 27)  + (25) |
|  |  | *Duration of the treatment* | N (5, 13)  - (10) |
|  | **Health system-related factors** | *Specialistic care* | *N (12)* |
|  |  | *Personalized care plan* | *+* (19, 25, 45). |
| **Environment** | **Community factors** | *Living in urban areas* | - (25)  + (27)  + (20) |
|  | **Social factors** | *Support from others* | *-(35)* |
|  | **Financial factors** | *Insurance status* | *+ (12, 22, 29)* |
|  |  | *Copayments* | *-(5)* |
|  |  | *Annual Income* | Annual household income that exceeded $70,000 + (3) |
|  |  | *Low Income subsidy* | *(9, 13, 46, 47)* |
|  |  | *Out-of-pocket cost* | *N (2)*  Higher patient out-of-pocket costs (5, 9, 20, 22, 25) |
|  |  | *Drug coverage* | *+(3)* |
| N: No significant; +: high adherence; -: low adherence | | | |

1. Ali EE, Cheung KL, Lee CP, Leow JL, Yap KY, Chew L. Prevalence and Determinants of Adherence to Oral Adjuvant Endocrine Therapy among Breast Cancer Patients in Singapore. Asia-Pacific journal of oncology nursing. 2017;4(4):283-9.

2. Bender CM, Gentry AL, Brufsky AM, Casillo FE, Cohen SM, Dailey MM, et al. Influence of patient and treatment factors on adherence to adjuvant endocrine therapy in breast cancer. Oncol Nurs Forum. 2014;41(3):274-85.

3. Bradley CJ, Dahman B, Jagsi R, Katz S, Hawley S. Prescription drug coverage: implications for hormonal therapy adherence in women diagnosed with breast cancer. BREAST CANCER RESEARCH AND TREATMENT. 2015;154(2):417-22.

4. Brett J, Fenlon D, Boulton M, Hulbert-Williams NJ, Walter FM, Donnelly P, et al. Factors associated with intentional and unintentional non-adherence to adjuvant endocrine therapy following breast cancer. EUROPEAN JOURNAL OF CANCER CARE. 2018;27(1).

5. Chin AL, Bentley JP, Pollom EL. The impact of state parity laws on copayments for and adherence to oral endocrine therapy for breast cancer. CANCER. 2019;125(3):374-81.

6. Corter AL, Broom R, Porter D, Harvey V, Findlay M. Predicting nonadherence to adjuvant endocrine therapy in women with early stage breast cancer. PSYCHO-ONCOLOGY. 2018;27(9):2096-103.

7. Font R, Espinas JA, Layos L, Villacampa MM, Capdevila J, Tobena M, et al. Adherence to capecitabine in preoperative treatment of stage II and III rectal cancer: do we need to worry? ANNALS OF ONCOLOGY. 2017;28(4):831-5.

8. Hefner J, Berberich S, Lanvers E, Sanning M, Steimer AK, Kunzmann V. Patient doctor relationship and adherence to capecitabine in outpatients of a German comprehensive cancer center. PATIENT PREFERENCE AND ADHERENCE. 2018;12:1875-87.

9. Hess LM, Louder A, Winfree K, Zhu YE, Oton AB, Nair R. Factors Associated with Adherence to and Treatment Duration of Erlotinib Among Patients with Non-Small Cell Lung Cancer. J Manag Care Spec Pharm. 2017;23(6):643-52.

10. Hwang GS, Paranjpe R, Opsomer C, Lu K, Abajue U, Abughosh S, et al. Oral Endocrine Therapy Agent, Race/Ethnicity, and Time on Therapy Predict Adherence in Breast Cancer Patients in a Large Academic Institution. CLINICAL BREAST CANCER. 2020;20(6):520-6.

11. Iacorossi L, Gambalunga F, Fabi A, Giannarelli D, Facchinetti G, Piredda M, et al. Adherence to hormone therapy in women with breast cancer: a quantitative study. Professioni infermieristiche. 2016;69(4):113-21.

12. Karavites LC, Kane AK, Zaveri S, Xu YF, Helenowski I, Hansen N, et al. Tamoxifen Acceptance and Adherence among Patients with Ductal Carcinoma In Situ (DCIS) Treated in a Multidisciplinary Setting. CANCER PREVENTION RESEARCH. 2017;10(7):389-97.

13. Neuner JM, Kamaraju S, Charlson JA, Wozniak EM, Smith EC, Biggers A, et al. The Introduction of Generic Aromatase Inhibitors and Treatment Adherence Among Medicare D Enrollees. JNCI-JOURNAL OF THE NATIONAL CANCER INSTITUTE. 2015;107(8).

14. Pan YQ, Heisig SR, von Blanckenburg P, Albert US, Hadji P, Rief W, et al. Facilitating adherence to endocrine therapy in breast cancer: stability and predictive power of treatment expectations in a 2-year prospective study. BREAST CANCER RESEARCH AND TREATMENT. 2018;168(3):667-77.

15. Stahlschmidt R, Ferracini AC, de Souza CM, de Medeiros LM, Juliato CRT, Mazzola PG. Adherence and quality of life in women with breast cancer being treated with oral hormone therapy. Supportive care in cancer : official journal of the Multinational Association of Supportive Care in Cancer. 2019;27(10):3799-804.

16. Sugita K, Kawakami K, Yokokawa T, Sugisaki T, Takiguchi T, Aoyama T, et al. Self-Reported Adherence to Trifluridine and Tipiracil Hydrochloride for Metastatic Colorectal Cancer: A Retrospective Cohort Study. ONCOLOGY. 2016;91(4):224-30.

17. Vacher L, Thivat E, Poirier C, Mouret-Reynier MA, Chollet P, Devaud H, et al. Improvement in adherence to Capecitabine and Lapatinib by way of a therapeutic education program. SUPPORTIVE CARE IN CANCER. 2020;28(7):3313-22.

18. Valachis A, Garmo H, Weinman J, Fredriksson I, Ahlgren J, Sund M, et al. Effect of selective serotonin reuptake inhibitors use on endocrine therapy adherence and breast cancer mortality: a population-based study. Breast Cancer Res Treat. 2016;159(2):293-303.

19. Blanchette PS, Lam M, Richard L, Allen B, Shariff SZ, Vandenberg T, et al. Factors associated with endocrine therapy adherence among post-menopausal women treated for early-stage breast cancer in Ontario, Canada. Breast Cancer Res Treat. 2020;179(1):217-27.

20. Dinan MA, Wilson LE, Greiner MA, Spees LP, Pritchard JE, Zhang T, et al. Oral Anticancer Agent (OAA) Adherence and Survival in Elderly Patients With Metastatic Renal Cell Carcinoma (mRCC). Urology. 2022;168:129-36.

21. Keim-Malpass J, Anderson RT, Balkrishnan R, Desai RP, Showalter SL. Evaluating the Long-Term Impact of a Cooperative Group Trial on Radiation Use and Adjuvant Endocrine Therapy Adherence Among Older Women. ANNALS OF SURGICAL ONCOLOGY. 2020;27(9):3458-65.

22. Pilon D, LaMori J, Rossi C, Durkin M, Ghelerter I, Ke X, et al. Medication adherence among patients with advanced prostate cancer using oral therapies. Future Oncology. 2022;18(2):231-43.

23. Pourcelot C, Orillard E, Nallet G, Dirand C, Billion-Rey F, Barbier G, et al. Adjuvant hormonal therapy for early breast cancer: an epidemiologic study of medication adherence. Breast Cancer Res Treat. 2018;169(1):153-62.

24. Haskins CB, McDowell BD, Carnahan RM, Fiedorowicz JG, Wallace RB, Smith BJ, et al. Impact of preexisting mental illness on breast cancer endocrine therapy adherence. Breast Cancer Res Treat. 2019;174(1):197-208.

25. Haskins CB, Neuner JM, McDowell BD, Carnahan RM, Fiedorowicz JG, Wallace RB, et al. Effects of Previous Medication Regimen Factors and Bipolar and Psychotic Disorders on Breast Cancer Endocrine Therapy Adherence. Clin Breast Cancer. 2020;20(3):e261-e80.

26. McGuinness S, Hughes L, Moss-Morris R, Hunter M, Norton S, Moon Z. Adherence to adjuvant endocrine therapy among White British and ethnic minority breast cancer survivors in the United Kingdom. European journal of cancer care. 2022;31(6):e13722.

27. Oke O, Niu J, Chavez-MacGregor M, Zhao H, Giordano SH. Adjuvant tamoxifen adherence in men with early-stage breast cancer. Cancer. 2022;128(1):59-64.

28. Tinari N, Fanizza C, Romero M, Gambale E, Moscetti L, Vaccaro A, et al. Identification of subgroups of early breast cancer patients at high risk of nonadherence to adjuvant hormone therapy: results of an Italian survey. Clin Breast Cancer. 2015;15(2):e131-7.

29. Yan YD, Fu J, Gu ZC, Lu JS, Su YJ, Lin HW. Adherence to endocrine therapy in patients with hormone receptor-positive early-stage breast cancer: a retrospective study. Int J Clin Pharm. 2023;45(1):184-90.

30. Zahrina AK, Norsa'adah B, Hassan NB, Norazwany Y, Norhayati I, Roslan MH, et al. Adherence to capecitabine treatment and contributing factors among cancer patients in Malaysia. Asian Pac J Cancer Prev. 2014;15(21):9225-32.

31. Hu X, Walker MS, Stepanski E, Kaplan CM, Martin MY, Vidal GA, et al. Racial Differences in Patient-Reported Symptoms and Adherence to Adjuvant Endocrine Therapy Among Women With Early-Stage, Hormone Receptor-Positive Breast Cancer. JAMA Netw Open. 2022;5(8):e2225485.

32. Murphy CC, Fullington HM, Gerber DE, Bowman IA, Puligandla M, Dutcher JP, et al. Adherence to oral therapies among patients with renal cell carcinoma: Post hoc analysis of the ECOG‐ACRIN E2805 trial. Cancer Medicine. 2021;10(17):5917-24.

33. Yuan C, Xie Z, Bian J, Huo J, Daily K. Outcomes of primary endocrine therapy in elderly women with stage I-III breast cancer: a SEER database analysis. Breast Cancer Res Treat. 2020;180(3):819-27.

34. Hirao C, Mikoshiba N, Shibuta T, Yamahana R, Kawakami A, Tateishi R, et al. Adherence to oral chemotherapy medications among gastroenterological cancer patients visiting an outpatient clinic. JAPANESE JOURNAL OF CLINICAL ONCOLOGY. 2017;47(9):786-94.

35. Bourmaud A, Henin E, Tinquaut F, Regnier V, Hamant C, Colomban O, et al. Adherence to oral anticancer chemotherapy: What influences patients' over or non-adherence? Analysis of the OCTO study through quantitative-qualitative methods. BMC Res Notes. 2015;8:291-.

36. Bright EE, Petrie KJ, Partridge AH, Stanton AL. Barriers to and facilitative processes of endocrine therapy adherence among women with breast cancer. Breast Cancer Res Treat. 2016;158(2):243-51.

37. Cakmak HSG, Uncu D. Relationship between Health Literacy and Medication Adherence of Turkish Cancer Patients Receiving Oral Chemotherapy. Asia-Pacific journal of oncology nursing. 2020;7(4):365-9.

38. Yang R, Lu Z, Gu X, Dai B. The Effect of an Information Support Program on Self-Efficacy of Prostate Cancer Patients during Hormonal Therapy. Asia-Pacific Journal of Oncology Nursing. 2021;8(6):639-52.

39. Koni AA, Suwan BA, Nazzal MA, Sleem A, Daifallah A, Allah MH, et al. Adherence to oral anticancer hormonal therapy in breast cancer patients and its relationship with treatment satisfaction: an important insight from a developing country. BMC Womens Health. 2023;23(1):114.

40. Rassy E, Bardet A, Bougacha O, Gantzer L, Lekens B, Delaloge S, et al. Association of Adherence to Endocrine Therapy Among Patients With Breast Cancer and Potential Drug-Drug Interactions. JAMA NETWORK OPEN. 2022;5(12).

41. Neugut AI, Zhong X, Wright JD, Accordino M, Yang J, Hershman DL. Nonadherence to Medications for Chronic Conditions and Nonadherence to Adjuvant Hormonal Therapy in Women With Breast Cancer. JAMA Oncol. 2016;2(10):1326-32.

42. Kawakami K, Nakamoto E, Yokokawa T, Sugita K, Mae Y, Hagino A, et al. Patients' self-reported adherence to capecitabine on XELOX treatment in metastatic colorectal cancer: findings from a retrospective cohort analysis. PATIENT PREFERENCE AND ADHERENCE. 2015;9:561-7.

43. Murphy CC, Fullington HM, Gerber DE, Bowman IA, Puligandla M, Dutcher JP, et al. Adherence to oral therapies among patients with renal cell carcinoma: Post hoc analysis of the ECOG-ACRIN E2805 trial. CANCER MEDICINE.

44. Zeidan B, Anderson K, Peiris L, Rainsbury D, Laws S. The impact of tamoxifen brand switch on side effects and patient compliance in hormone receptor positive breast cancer patients. Breast. 2016;29:62-7.

45. Arriola KRJ, Mason TA, Bannon KA, Holmes C, Powell CL, Horne K, et al. Modifiable risk factors for adherence to adjuvant endocrine therapy among breast cancer patients. PATIENT EDUCATION AND COUNSELING. 2014;95(1):98-103.

46. Ma SY, Shepard DS, Ritter GA, Martell RE, Thomas CP. The impact of the introduction of generic aromatase inhibitors on adherence to hormonal therapy over the full course of 5-year treatment for breast cancer. CANCER. 2020;126(15):3417-25.

47. Winn AN, Fergestrom NM, Neuner JM. Using Group-based Trajectory Models and Propensity Score Weighting to Detect Heterogeneous Treatment Effects The Case Study of Generic Hormonal Therapy for Women With Breast Cancer. MEDICAL CARE. 2019;57(1):85-93.
